# Lead radionuclides for theranostic applications in nuclear medicine: from atom to bedside

**DOI:** 10.7150/thno.126086

**Published:** 2026-01-01

**Authors:** Yani Berckmans, Janke Kleynhans, Sara Van Mechelen, Karolien Goffin, Kristof Baete, Michel Koole, An Coosemans, Thomas Elias Cocolios, Christophe M Deroose, Yann Seimbille, Frederik Cleeren

**Affiliations:** 1 KU Leuven, Department of Oncology, Laboratory for Tumor Immunology and Immunotherapy, Leuven Cancer Institute, Leuven, 3000, Belgium.; 2 KU Leuven, Department of Pharmaceutical and Pharmacological Sciences, Laboratory for Radiopharmaceutical Research, Leuven, 3000, Belgium.; 3 Belgian Nuclear Research Centre (SCK CEN), Radiobiology Unit, Nuclear Medical Applications Institute, Mol, 2400, Belgium.; 4 KU Leuven, Department of Imaging and Pathology, Nuclear Medicine and Molecular Imaging, Leuven, 3000, Belgium.; 5 KU Leuven, Department of Physics and Astronomy, Institute for Nuclear and Radiation Physics, Leuven, 3001, Belgium.; 6 Department of Radiology and Nuclear Medicine, Erasmus MC, 3015 CN Rotterdam, The Netherlands.; 7 Erasmus Medical Center Cancer Institute, 3015 GD Rotterdam, The Netherlands.

**Keywords:** lead-212, lead-203, radionuclide therapy (RNT), targeted radionuclide therapy (TRT), targeted alpha therapy (TAT), single-photon emission tomography (SPECT)

## Abstract

Lead-212 has emerged as a promising radionuclide for targeted alpha therapy (TAT), positioning itself at the forefront of next-generation cancer treatments. What sets lead-212 apart is its unique decay profile: while it is a beta emitter, it mainly serves as an *in vivo* generator of the potent alpha-emitting daughter radionuclide bismuth-212. This characteristic offers a versatile radiobiological profile, potentially optimizing therapeutic efficacy by combining the radiochemical characteristics of lead-212 such as easy radiolabeling and practical half-life, with the therapeutic benefit of alpha particles. The relatively short half-life of lead-212 (10.6 hours) offers favorable dosimetric properties, enabling effective treatment while minimizing long-term radiation exposure. Its gamma-emitting analogue, lead-203, is well-suited for single-photon emission computed tomography (SPECT) imaging, forming an ideal theranostic matched pair that can significantly accelerate preclinical and clinical research. Crucially, the generator-based production of lead-212 enables decentralized and on-demand availability, but its half-life also allows centralized production, facilitating broad clinical access and logistical flexibility. In this review, the advantages and challenges of lead-based radiopharmaceuticals are discussed, covering the entire chain from radionuclide production to bedside administration. Special attention is given to the coordination chemistry of lead, and we give an overview of available bifunctional chelators. We also present the latest advancements in preclinical and clinical applications and conclude with perspectives on future directions of lead-based theranostics.

## Introduction

As the global pursuit of precision cancer therapies accelerates, alpha-emitting radionuclides are gaining increased attention for use in radionuclide therapy (RNT), although ensuring sustainable and widespread access to these powerful agents remains a critical challenge 1. Lead-212, with its generator-based production methods and alpha-emitting decay chain, offers a unique solution to these supply constraints and could pave the way for a broader clinical adoption of targeted alpha therapy (TAT) 2. The raw materials required to produce lead-212, such as thorium-228 derived from legacy nuclear sources, are reported to be in ample supply 3. Furthermore, the dual functionality offered by the lead-203/lead-212 theranostic pair will allow for the quantification of the biological behavior of the radionuclide during the translational phase 4.

Historically, diagnostics and therapeutics in medicine have evolved as separate disciplines, often operating in parallel rather than synergistically. Nuclear medicine, however, offers a distinct advantage through the concept of *theranostics*, which aligns closely with the principles of precision medicine. This approach enables both diagnosis and treatment using the same molecular target. Diagnosis is typically achieved through non-invasive molecular imaging techniques such as positron emission tomography (PET) or single-photon emission computed tomography (SPECT), which first confirms that a specific biological target is present in sufficient quantity within the tumor. Following initial identification, the diagnostic non-cytotoxic radionuclide may be exchanged for a therapeutical cytotoxic analogue, utilizing an analogous molecular vector. This theranostic approach allows selective irradiation of the malignancy depicted in the initial molecular imaging scan 5. Prominent examples of theranostics include targeting prostate-specific membrane antigen (PSMA) in castration-resistant prostate cancer, where the therapeutic radiopharmaceutical is [^177^Lu]Lu-PSMA-617 (United States Food and Drug Administration (FDA) approved as Pluvicto® in 2022), and somatostatin receptors (SSTRs) in neuroendocrine tumors (NETs), with [^177^Lu]Lu-DOTA-TATE (FDA approved as Lutathera® in 2018) as the therapeutic partner 6-8. In these cases, diagnostic radiopharmaceuticals using PET radionuclides such as gallium-68 or fluorine-18 can serve as the diagnostic counterparts 6-8.

While these beta-minus (beta-) emitting theranostic approaches have demonstrated substantial clinical benefit, they are not without limitations. The relatively low linear energy transfer (LET) of beta-particles is still insufficient to deliver a lethal dose to the tumor cells in current clinical applications. Their cytotoxicity therefore relies heavily on the presence of oxygen to generate reactive oxygen species. Additionally, resistance can arise through mechanisms such as upregulated DNA repair pathways and reduced sensitivity to radiation-induced DNA damage 9. In contrast, alpha particles are highly cytotoxic due to their high LET, resulting in the deposition of energy over a very short path length in biological tissue, typically spanning just 2 to 10 cell diameters. This concentrated energy delivery causes dense ionization tracks that lead to complex and clustered DNA damage, including double-stranded DNA breaks, which are often difficult to accurately repair, increasing the likelihood of lethal outcomes such as apoptosis or mitotic catastrophe. Moreover, in hypoxic conditions, alpha emitters maintain their effectiveness due to their direct ionization mechanism, making them less dependent on the oxidative status of malignant lesions 9,10. An additional benefit of alpha emitters is the amplified cytotoxicity through effective activation of the immune system which might incorporate bystander and other systemic responses. TAT is consequently highly effective against hypoxic and radioresistant disease, both in micro-metastases as well as larger tumors 9,10.

Despite encouraging initial clinical outcomes, the search for the optimal alpha-emitting radionuclide remains ongoing. When the respective nuclear properties are considered, eight alpha emitters have been identified as promising potential candidates for clinical translation 11. However, many of these radionuclides face significant production and supply constraints. Therefore, the selection cannot rely solely on favorable nuclear characteristics, such as physical half-life, emission profile, and chemical properties, or on biological factors like radiobiological efficacy. Consequently, the field has largely narrowed its focus to three alpha emitters with more feasible production pathways: astatine-211, actinium-225, and lead-212 3,12.

Lead-212 is a particularly promising candidate for TAT as previously also discussed in a review by Scaffidi-Muta and Abell 13. Due to its unique decay profile, lead-212 functions both as a beta emitter and as an *in vivo* generator of the alpha-emitting daughter nuclide bismuth-212, effectively delivering cytotoxic alpha radiation at the tumor site. Its relatively short physical half-life offers favorable irradiation characteristics, potentially reducing off-target radiation exposure when combined with a suitable vector molecule and improving therapeutic index compared to longer-lived alpha emitters 3. An additional advantage of lead-212 is the availability of its chemically identical isotope, lead-203, a gamma emitter suitable for SPECT imaging. While its usefulness in the everyday clinical setting might yet need to be established as more convenient PET alternatives exist, having a radioisotope that can be imaged and quantified during clinical trials and research and development remains an advantage above most other therapeutic radionuclides.

In this review, we provide a comprehensive overview of the potential of lead-212 for TAT, covering the full translational pipeline from isotope production to clinical application. We begin by discussing the physical and chemical properties of lead-212 and its imaging counterpart, lead-203, emphasizing their compatibility for theranostic use. This includes a detailed examination of available production methods, with a focus on generator-based systems and the feasibility of large-scale supply. We then explore radiolabeling strategies and the coordination chemistry of lead isotopes, including a review of bifunctional chelators suited for stable complexation. Key considerations in designing lead-based radiopharmaceuticals are addressed, followed by a summary of current preclinical and early clinical studies, including data on biodistribution, dosimetry, therapeutic efficacy, and toxicity. Finally, we outline the challenges and future directions for integrating lead-212 into routine clinical practice, highlighting its promise as a scalable and effective alpha-emitting therapeutic in nuclear medicine.

## Radionuclide Properties and Production Methods of Lead-212 and Lead-203

### Decay properties

#### Lead-212

**Figure 1:**
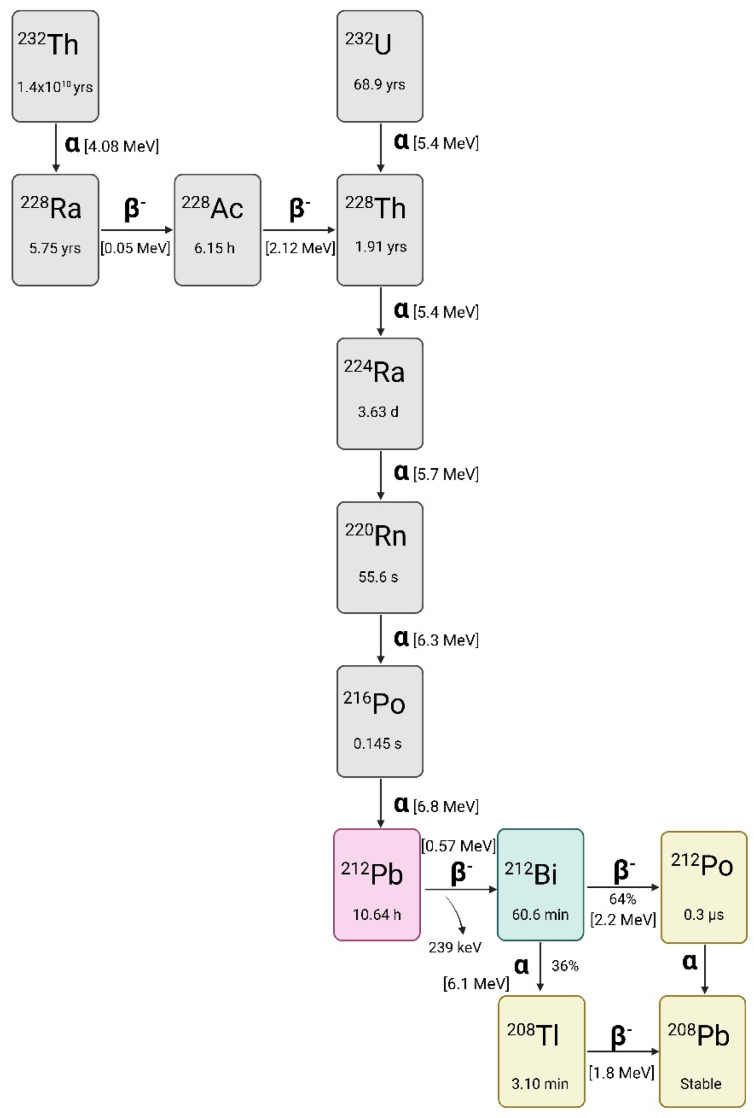
The decay chain of thorium-232 and uranium-232 as the source for lead-212, drawn with a licensed version of BioRender.com 22.

Figure 1 displays the full decay scheme of lead-212, which is part of the decay chain of the long-lived radionuclides uranium-232 (t_1/2_ = 68.9 years) and thorium-232 (t_1/2_ = 1.04 x10^10^ years) 1​. With a physical half-life of 10.64 hours, lead-212 decays 100% through β^-^ emission (E_β-_ = 0.57 MeV) to bismuth-212 (t_1/2_ = 60.5 min). Using lead-212 as an *in vivo* generator over the direct administration of bismuth-212 is hypothesized to have significant clinical advantages. Most notably, the longer half-life can lead to a ten-fold reduction in activity per patient administration compared to bismuth-212 alone 12.

The daughter radionuclide bismuth-212 decays via two paths, both resulting in an emission of one high-energy cytotoxic alpha particle. One decay path of bismuth-212 is through direct alpha particle emission for 35.93% to thallium-208 (t_1/2_ = 3.06 min) with the residual 64.07% of bismuth-212 decaying to polonium-212 (t_1/2_ = 0.3 µs) via another β^-^ emission (E_β-_ = 1.8 MeV). Polonium-212 is a pure alpha emitter and decays to stable lead-208. Lastly, thallium-208 decays via both beta particle and gamma emission to stable lead-208 1,3,14.

During the decay of lead-212, both X- and gamma rays are emitted, allowing for convenient radioactivity quantification 15. The principal gamma-line used for measurement of lead-212 is 238.6 keV (43.6%) while several lower energy X-rays such as 74.8 keV (0.28%), 77.1 keV (17.1%), 86.8 keV (2.07%), 87.3 keV (3.97%) and 89.8 keV (1.46%) can also be exploited for gamma-spectrometry using sodium iodide (NaI) detectors 16. However, the daughter radionuclides bismuth-212 and thallium-208 emit additional X- and gamma-radiation during their decay. Therefore, to minimize interference from these progenies, it can be advantageous to restrict the measurement to a defined energy window (e.g. 60-110 keV) when using NaI detectors 16,17.

As is the case with other alpha-emitting radionuclides where unlabeled daughters must decay before quality control samples (TLC and HPLC) can be measured. In such cases, the true radiochemical purity of a ^212^Pb-radiopharmaceutical is viewed as the relationship between free lead-212 and the ^212^Pb-radiopharmaceutical, with no influence by the non-bound bismuth-212 that was present in the reaction mixture. For both TLC measurements and fractions collected from radio-HPLC, it is important to wait 3-4 hours for the final determination of radiochemical purity 18. Additionally, if concerns arise regarding the biodistribution of unbound bismuth-212 to the kidneys, DTPA in a concentration of 0.1 mg/mL can be added to provide renal protection.

When quantifying lead-212 using ionization chambers (dose calibrators), caution should be taken when using purified or freshly produced lead-212. Such lead-212 samples are devoid of daughters and subsequent ingrowth leads to time-dependent variability in the detected activity. Accurate measurements therefore require either the application of a correction factor or allowing sufficient time for equilibrium to be established (approx. six hours). Notably, predefined calibration factors or dial settings provided by the manufacturers are generally estimated for isolated lead-212, without accounting for daughter radionuclides. Since ingrown daughters such as bismuth-212 and thalium-208 contribute substantially to the dose calibrators' response, standardization or calibration of the measurement practices should be performed before studies are initiated 17. International efforts are currently trying to standardize activity measurements of lead-212 through the implementation of appropriate calibration protocols.

Several studies have also explored the use of SPECT/CT for lead-212 and proposed some acquisition protocols that could be useful for image quantification 19-21. It must be mentioned that lead-212 releases a high-energy gamma ray (2.6 MeV) during decay originating from its daughter radionuclide thallium-208. This necessitates the implementation of additional safety measures for healthcare personnel and researchers working with this radionuclide 1,3.

The preparation of sources containing lead-203 or lead-212 for the calibration or verification of medical imaging equipment can require large amounts of fluids. Plastic phantoms that are used for these devices, such as uniform cylinders, require liquids in which the radioactive substance is expected to be uniformly dissolved. The chemical form in which the radioactivity is present will be crucial for the calibration or verification steps. Therefore, the radiochemical form must be considered, and care must be taken such that the diluted substance remains uniform and does not adhere or precipitates to the walls of the recipient or phantom during the entire procedure.

#### Lead-203

**Figure 2:**
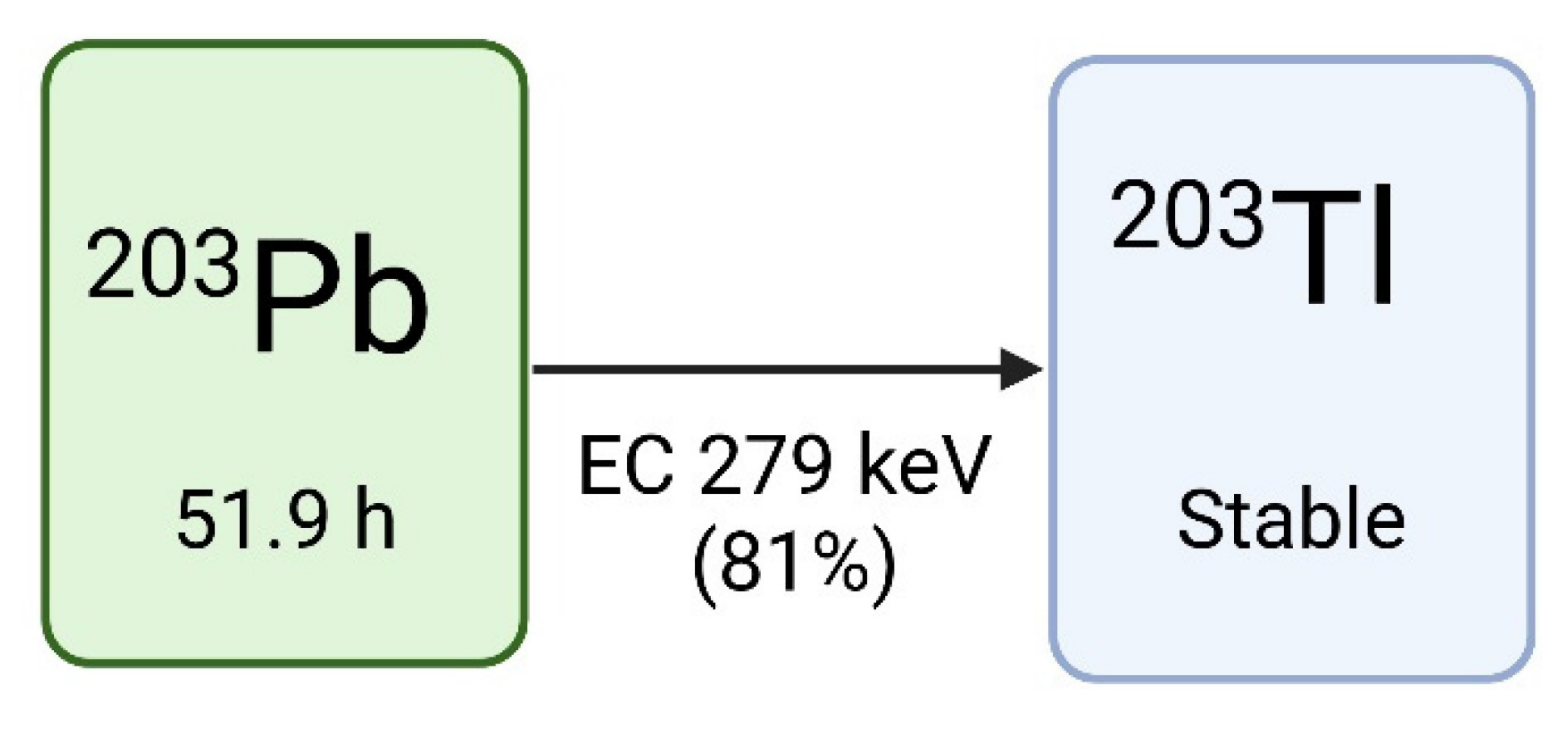
the decay scheme of lead-203, drawn with a licensed version of BioRender.com 28.

Lead-203 has a half-life of 51.9 hours and decays to stable thallium-203 via electron capture (EC), resulting in the release of a gamma photon (279 keV, Iγ=81%) compatible with SPECT imaging (Figure 2). This low-energy gamma photon, in addition to the residual 401 keV (5%) and 680 keV (0.9%) gamma energies, permits imaging using both a low and high energy SPECT collimator 4,23-26. Due to the relatively long half-life of lead-203, extended imaging is feasible to gather all necessary information needed for lead-212 treatment dosimetry 4. The theranostic pairing of lead-203 and lead-212 is compelling due to their identical chemical characteristics, which warrants consistent biodistribution profiles 27. However, it must be noted that lead-203 does not match the decay scheme (and changes in chemistry) of lead-212. Imaging using lead-203 can therefore not capture the daughter radionuclide decays (such as bismuth-212, which is the actual alpha emitter) which will have completely different biodistribution profiles if chelator dissociation occurs *in vivo*.

### Current strategies for production

#### Lead-212

**Figure 3:**
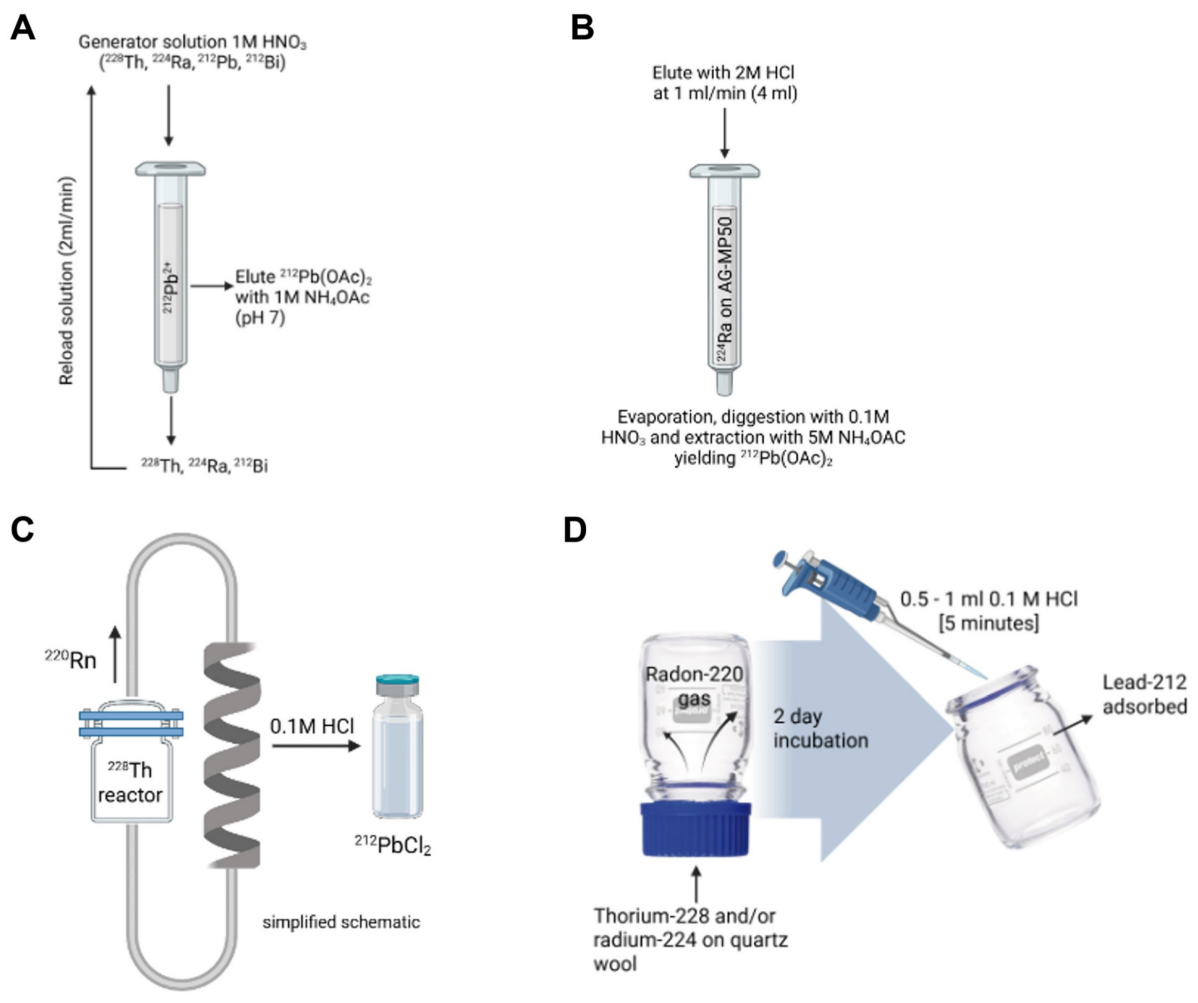
A and B) column generators based on respectively thorium 228 and radium-224 4,29, and C and D) examples of radon-220 gas emanation generators. Drawn with a licensed version of BioRender.com 2,28.

The production of lead-212 is based on generator systems separating lead-212 from decaying parent radionuclides thorium-228 or radium-224. Lead-212 generators reported in literature describe the separation of daughter radionuclides through either a cation exchange column loaded with thorium-228 (Figure 3A) or radium-224 (Figure 3B) or emanation of the gaseous daughter radionuclide, radon-220 (Figure 3C and 3D) 4​. Many generators have been described, and some technical details are provided in Table 1.

##### Column generator systems based on thorium-228

The use of thorium-228-based lead-212 generators may hold some benefits such as a long half-life of 1.9 years limiting the generator replacement, reducing production cost, and lowering radiation dose to exposed personnel. A complex system was described early by Morimoto and Khan 30 through the electrodeposition and adsorption of ^228^Th-hydroxide on a positive aluminum plate. The lead-212 is moved through a potential difference to a negatively charged platinum coil, which is then dissolved after deposition. However, this method yielded very low yields of approximately 20%.

A first ^228^Th/^212^Pb column generator was introduced by Zucchini and Friedman in 1982, consisting of a novel two-step system based on the cation exchange principle. In the first column, both thorium-228 and radium-224 are absorbed on Na_2_TiO_3_. Radon-220 is eluted before loading onto a second column containing D-50 cation exchange resin, and from this second column, lead-212 could be eluted using 2M HCl with a maximal yield of 85%. However, small amounts of thorium-228 and radium-224 breakthrough were noted 31​. In a more advanced system, McAlister and Horwitz described a lead-212-generator from thorium-228 as parent radionuclide, using a three-cartridge system of UTEVA resin (retaining thorium-228), Sr resin (capturing lead-212), and a prefilter resin to remove extractant traces. Radium-224 is not retained by this system. Afterwards, lead-212 is recovered with a high yield of > 95% 32​.

##### Column generator systems based on Radium-224

Upscaling of thorium-228 based generators to produce clinical amounts of activity is found problematic due to radiolytic damage to the columns 33,34​. To circumvent this problem,^ 224^Ra-based column generators are being investigated. These generators have a much shorter shelf life due to the parent half-life of 3.6 days. A crucial step is the complete separation of radium-224 from thorium-228. Atcher *et al.* described the use of anion exchange columns early on 35. The pure radium-224 can then be loaded on a cartridge system (for example, a cation exchange resin) to create a generator from which lead-212 can be eluted using an acidic medium 28,35-39. It is important to note that this generator system has been in use now for more than 30 years and is supplied by OranoMed and Oak Ridge National Laboratory 34. However, the use of additional purification columns has been suggested to remove all traces of the radium-224 from the lead-212 eluate and ensure no breakthrough 40,41​.

##### Emanation generators based on capturing gaseous radon-220

The principle of radon-220 emanation for use in lead-212 production was described early on by Gregory *et al.* 42 using the parent radionuclide thorium-228. In such systems, radon-220 is trapped in a secondary chamber after emanation from either the parent radionuclide thorium-228 or radium-224. Lead-212 is subsequently collected after radon-220 decay. Similarly, a ^228^Th/^212^Pb-generator system based on this principle was patented by Norman *et al.* in 1991, where decayed radon-220 gas from thorium-228 placed in one chamber is diffused to a second chamber, after which lead-212 may be collected 43.

Hassfjell and Hoff reported a ^228^Th/^212^Pb-generator using a different collection principle in 1994, where a [^228^Th]barium stearate source was placed in an air-tight collection chamber to increase the diffusion distance of radon-220 gas. Following the deposition of lead-212 on the collection chamber walls, this could be collected using distilled water 44. Later, this generator was adapted to produce increased levels of radioactivity, closer to clinically relevant amounts, by collecting radon-220 emanating from [^228^Th]barium stearate in a glass bubbler filled with an organic solvent at -72 °C 33.

Boldyrev and co-workers introduced a helix-shaped collector vessel into their ^228^Th/^212^Pb-generator, in which emanating radon-220 is transported and the deposited lead-212 may be collected using 0.1M HCl 45. Li and co-workers 2 recently proposed a ^224^Ra/^212^Pb-generator based on this radon-220 emanation principle, similar to previous ^228^Th/^212^Pb-generator systems. However, the scalability of this system is lacking due to the use of a single chamber generator consisting of an inverted 100 mL glass flask containing radium-224 in the screw cap, pipetted onto quartz wool. After decay, lead-212 may be extracted from the interior of the glass flask 2. Recently, CERN developed a similar generator system, leveraging its high yields in extracting radium-224 from irradiated thorium-232 targets at the MEDICIS facility 46. Regular supply is now available, either directly from the MEDICIS facility or via the PRISMAP infrastructure 12.

##### Column generator systems vs emanation-based systems

Two main strategies for lead-212 based generators exist namely immobilizing the long-lived parent on a solid support that allows periodic elution, and emanation-based generators where radon-220 is collected in a vessel or chamber, after which lead-212 is collected subsequently by elution. Both options have advantages and disadvantages. In the column-based approach, the parent is fixed to the matrix and allows for a more robust and repeatable elution and minimal handling of parent radionuclides. Chromatographic column generators are more optimal for upscaling radiopharmaceutical production and easier to incorporate in automated production strategies. It is important to determine whether there could be radiolytic damage of the columns if thorium-228 is kept stored absorbed onto it as this can influence column efficiency and yield. On the other hand, emanation design physically separates the daughter radionuclides from the parent radionuclides, providing a very pure lead-212. This comes at the cost of lower yields and greater complexity in generator handling.

#### Alternative Lead-212 Sources and Radiochemical Processing

A system exploring the use of the parent radionuclide uranium-232 (deposited on a steel plate) was investigated in the past but was found unsuitable for medical purposes 49. Bartoś and co-workers also described the separation of lead-212 (1 MBq) from uranium-232 but did not present or evaluate their method as a generator system. Uranium-232 and decay daughters were loaded on an HDEHP-Teflon column, subsequently radium-224 was eluted by 0.1 M HNO_3_ and loaded on a cation exchange resin (Dowex 50 x 8). Lead-212 was then eluted with 0.1 M HCl for further processing 37.

Most generator systems yield large volumes of eluted lead-212, often in solvents incompatible for radiochemistry procedures. Therefore, additional evaporation may be required, increasing the chance of impurities and reducing the production yield 3. Currently, two systems, using respectively a thorium-228 and radium-224 based column generator, describe the final elution in an NH_4_OAc solution, which is directly compatible with radiolabeling 4,47. Another technology additionally added an anion exchange purification column to reduce metal impurities, such as Fe^3+^, without significant loss of lead-212 activity 23.

##### Availability and Supply of Lead-212

The development of lead-212 generator systems represents a dynamic and rapidly advancing field, driven by significant industrial and academic investment. Optimization of both column-based and emanation-type generators has demonstrated the feasibility of scaling-up the production to meet clinical demand. Moreover, both centralized and decentralized production of lead-212 is actively being pursued within the radiopharmaceutical industry, underscoring the versatility and potential of lead-212 supply strategies.

As for access to thorium-228 or radium-226, it is stated in a recent commentary by Zimmermann 3, that there are ample sources from legacy nuclear material that yield thorium-228. Thorium-228 as starting material can be produced by several methods. This includes the use of radium-226 as target material, spallation of thorium-232 or the isolation of thorium-228 from legacy stockpiles. For an in-depth discussion on the production methods of thorium-228 we refer the reader to previously published reviews 1,12,13. Since radium-226 is the main starting material to produce radium-223 and actinium-225, direct production of lead-212 is also deemed feasible from radium-226 via the ^226^Ra(γ,2n)^224^Ra reaction using a linear accelerator. Therefore, no shortage of lead-212 is expected.

**Figure 4:**
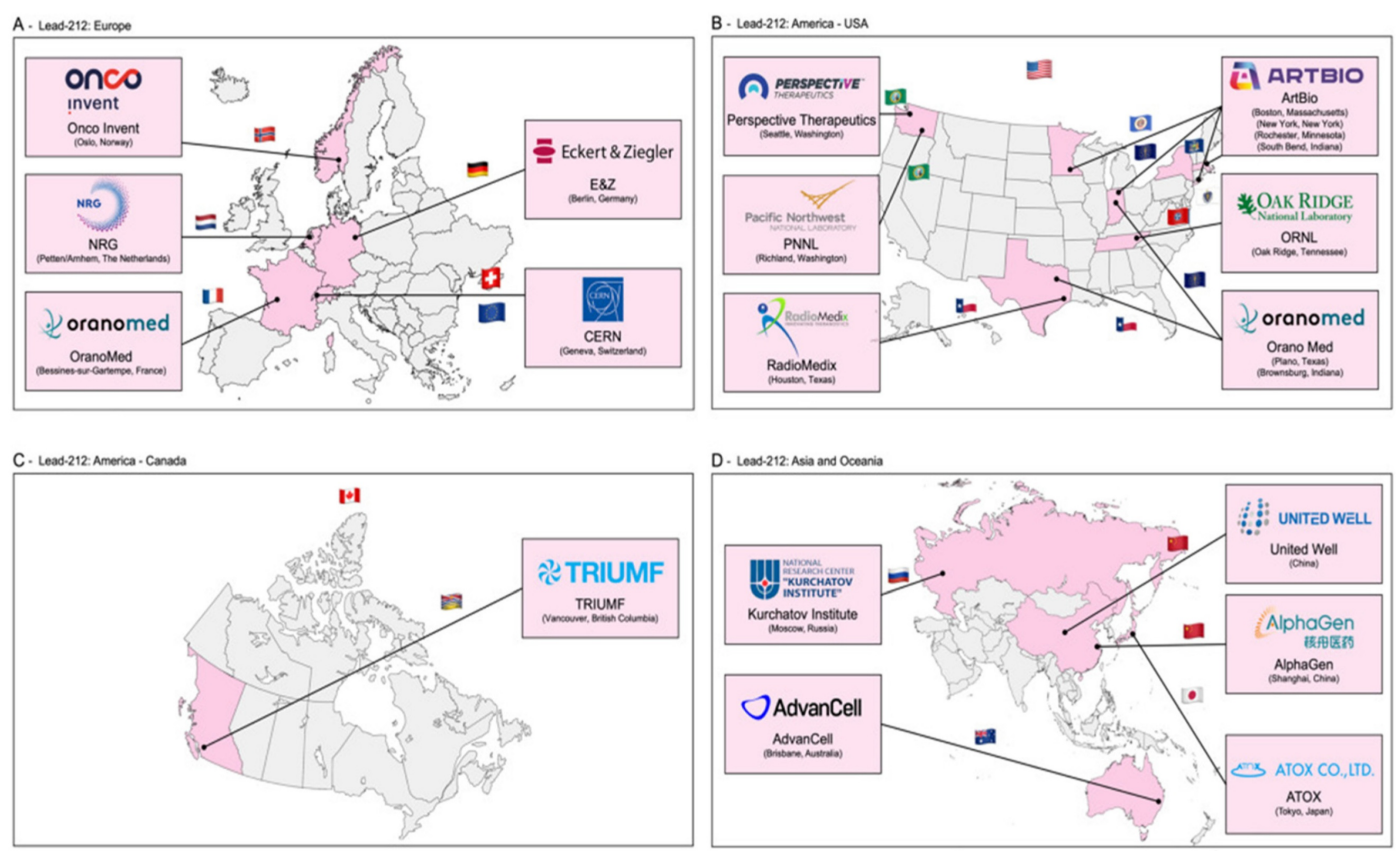
Geographical spread of sites that currently have experience in the production of lead-212 (reprinted with permission, 12).

In Figure 4, the recently published distribution of sites that have experience in the production of lead-212 is presented 12. Lead-212 is also available from suppliers (such as OranoMed) 50 in the form of^ 212^Pb(HNO_3_)_2_ as an eluate. Its half-life of 10.6 hours allows for distribution to other radiopharmaceutical sites 50,51.

#### Lead-203

##### Lead-203 cyclotron production

The cyclotron-based production of lead-203 can be achieved through charged particle bombardment of natural thallium consisting of 29.5% thallium-203 and 70.5% thallium-205 or isotopically enriched thallium 24-26​. Theoretically, lead-203 could also be produced through bombardment of enriched mercury, but this is considered too expensive 24. A low energy proton beam (e.g., 13 MeV) results in the production of lead-203 from the ^203^Tl(p,n)^203^Pb nuclear reaction using isotopically enriched thallium-203 52. Production yield is limited due to the low cross-section of this reaction at 11 MeV (111 mb) or 13 MeV (37.4 mb) and the low melting point of thallium (304 °C). This results in low thermal stability 25,26. Using this production technique, the use natural thallium is avoided due to its high fraction of thallium-205 which will result in the production of lead-205 (T_1/2_ 15.3 My) following the ^205^Tl(p,n)^205^Pb reaction, complicating the production process.

Alternatively, lead-203 may be produced using a proton energy beam above 17 MeV (e.g., 24 MeV) through the ^205^Tl(p, 3n)^203^Pb nuclear reaction, which is ideally performed using isotopically enriched thallium-205 to avoid the production of lead-201 from the ^203^Tl(p, 3n)^201^Pb nuclear reaction when using natural thallium 53​. In this reaction, a larger cross section was reached (σmax = 1193 mb at 28 MeV) using isotopically enriched thallium-205, resulting in a high yield of lead-203. When natural thallium is used as a target material, a suitable decay time of the ^201^Pb (t_1/2_ = 9.3 h) by-product is required before the purification of lead-203, since these radioisotopes cannot be separated efficiently 41​.

The production of lead-203 is also possible through deuteron bombardment of isotopically enriched thallium-203 from the ^203^Tl(d,2n)^203^Pb nuclear reaction. However, isotopically enriched thallium-203 is more expensive than thallium-205 due to its abundance in naturally occurring thallium 26​. The use of sealed solid target material, as described by Nelson *et al.*, further improves production yield by enabling target material cooling and preventing thallium oxidation 25​. Moreover, since enriched target materials are expensive, production methods aim to recycle the thallium to decrease production costs. This can be done through multiple methods such as chemical oxidation and precipitation as described by McNeil *et al.* 23.

**Figure 5:**
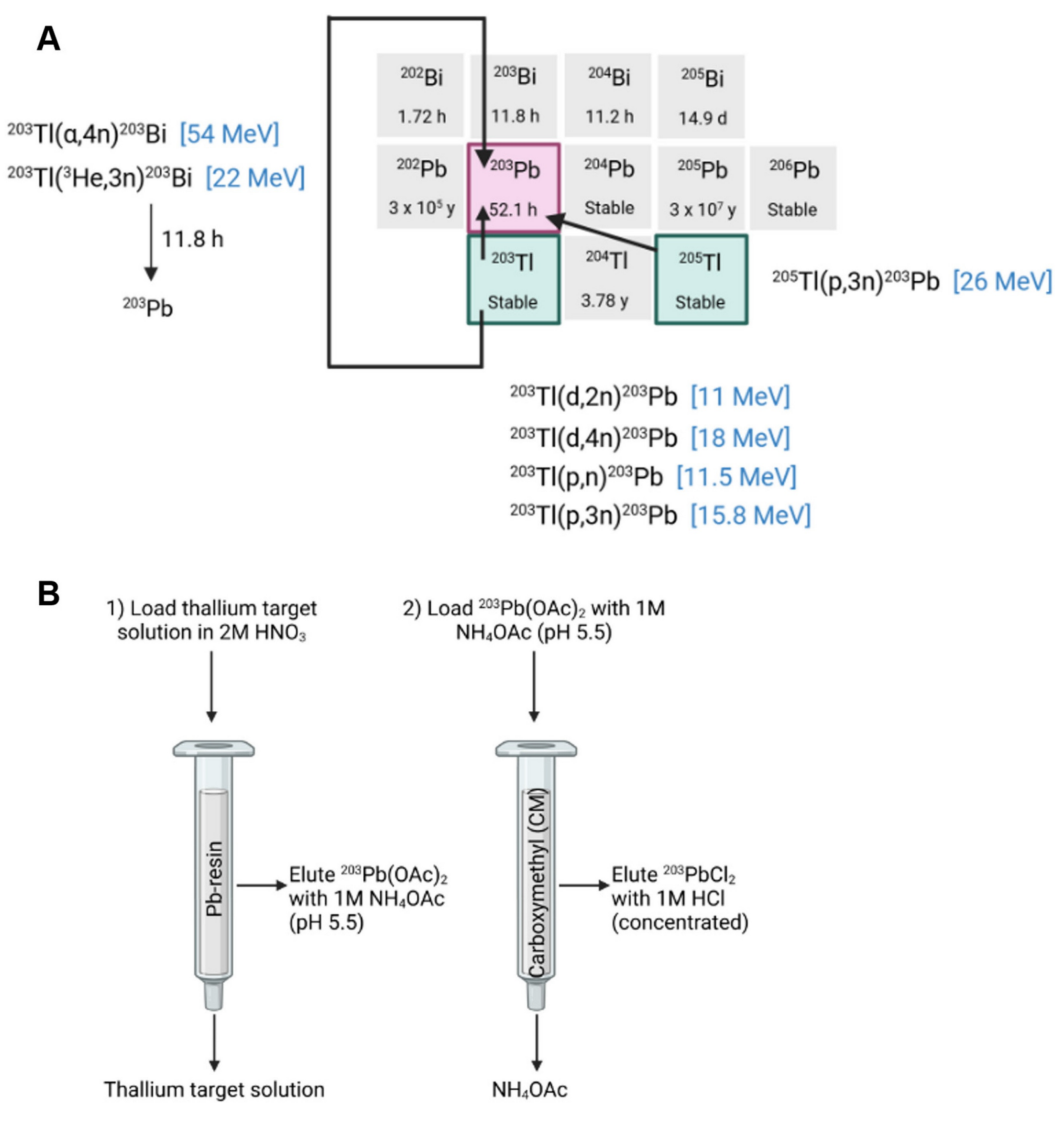
A) A summary of possible lead-203 production routes 24,53 B) An example of a separation scheme for purification of lead-203 from a thallium target, drawn with a licensed version of BioRender.com 54.

Logically, purification of lead-203 is similar to lead-212 purification methods developed and normally includes the application of anion exchange or Pb-selective resins. Again, these purification steps may result in large volumes of eluate requiring evaporation of the lead-203 eluates and re-dissolution before radiolabeling. Saini *et al.* described a robust purification method to produce high-purity lead-203 using a two-column separation method. The irradiated targets are first dissolved in HNO_3_ and loaded onto a Pb-selective resin. Residual thallium is removed from the resin bed by washing it with additional HNO_3_ before lead-203 is eluted as an NH_4_OAc solution. This eluate was then loaded onto a second cation exchange resin for concentration, from which lead-203 was eluted as [^203^Pb]PbCl_2_ using HCl. This allows for direct radiolabeling without evaporation 54. A summary of the production methods of lead-203 is provided in Figure 5.

##### Availability and Supply of Lead-203

The availability of lead-203 is limited to the available production facilities equipped with medium-energy cyclotrons and the access to enriched target material 3. However, growing interest in lead-203 as a diagnostic counterpart to lead-212 in theranostic applications has prompted increased efforts to improve supply chains and expand availability through both academic and industrial initiatives.

**Figure 6:**
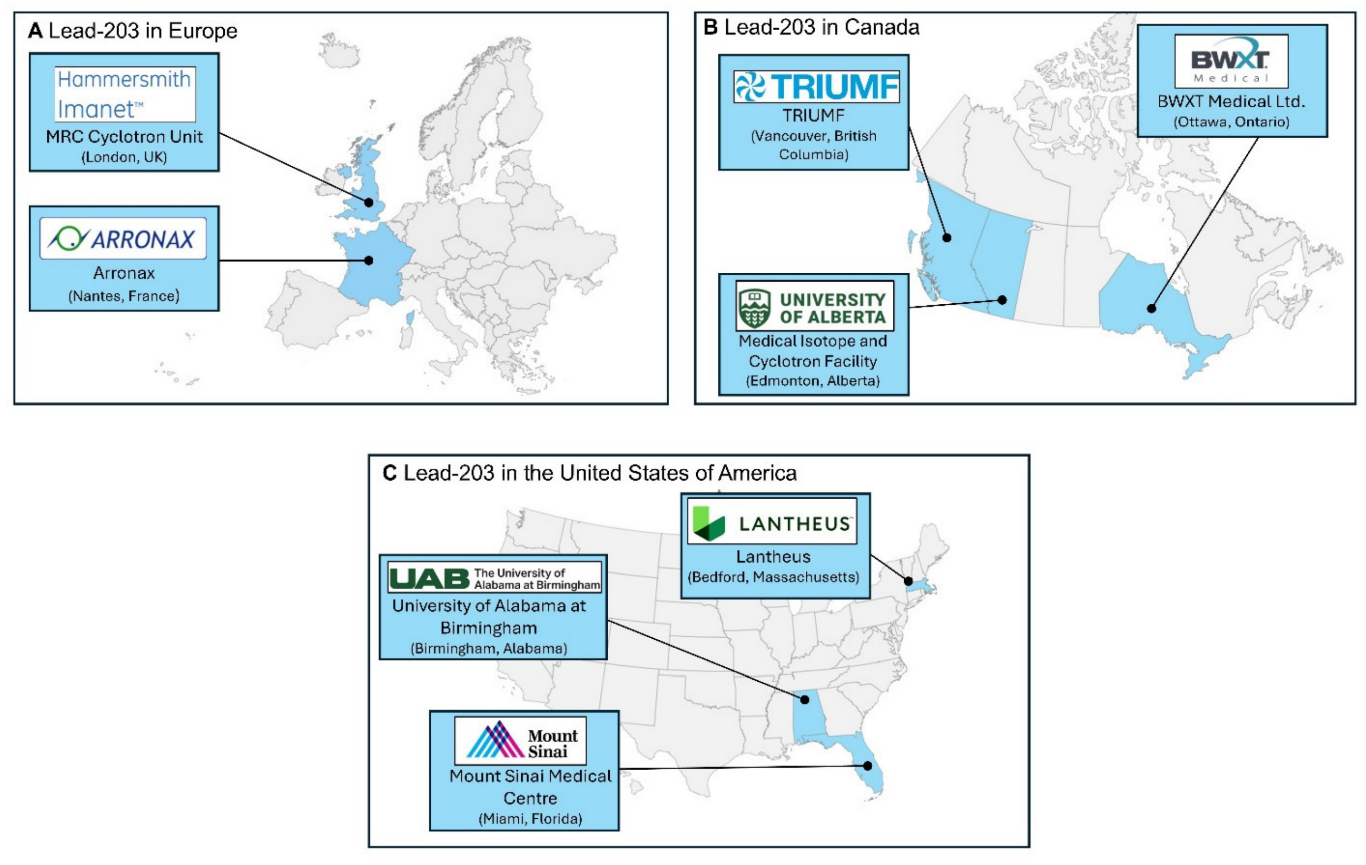
Geographical spread of sites that currently have experience in the production of lead-203 23,54-58.

In Figure 6, we provide a lead-203 production atlas, to highlight locations where lead-203 was obtained from in the past five years, reported in literature or via an online search for suppliers. The terms “producer” and “supplier” should not be interpreted literally in this context. This might not be an exhaustive list of sites that have experience in the production of lead-203 or are able to provide lead-203 for preclinical and/or clinical purposes.

## Chemical characteristics of lead

In aqueous solution at ambient conditions, lead exists predominantly as a divalent cation (Pb²⁺) with an ionic radius of 1.19 Å (coordination number of 4-12) 34,59,60. As a borderline cation, Pb²⁺ can interact with both hard donors (e.g., oxygen) and soft donors (e.g., sulfur) 60,61. This allows Pb²⁺ to form complexes with a wide range of stabilities and geometries.

Although the ionic radius of Pb²⁺ is similar to that of Sr²⁺ (1.18 Å), its coordination behavior is notably distinct due to relativistic effects. As a heavy element, lead experiences relativistic contraction of its 6s orbital, lowering its energy and reducing its participation in bonding, an effect known as the inert pair effect. This phenomenon contributes to the formation of asymmetric or hemidirected coordination complexes.

In contrast, Sr²⁺ lacks this inert pair effect and typically forms more rigid, symmetric complexes with a strong preference for hard donors like oxygen. Pb²⁺, on the other hand, exhibits greater flexibility in chelation due to its ability to coordinate with both hard and soft donors. This unique stereochemical behavior underlies the distinctive chelator preferences of Pb²⁺ and sets it apart from other divalent cations of similar size 34,59,62-65. The lone pair may either be stereochemically active, contributing to an asymmetric coordination environment (hemidirected geometry), or stereochemically inactive, resulting in a more symmetrical arrangement of ligands (holodirected geometry).

When the lone pair is active, it occupies one side of the coordination sphere, forcing ligands to arrange themselves on the opposite side, creating an asymmetrical (hemidirected) complex. In contrast, when the lone pair is inactive, ligands are distributed more evenly around the Pb²⁺ center, yielding a symmetrical (holodirected) complex. The stereochemical activity of the lone pair is strongly influenced by the nature and coordination number of the ligands. Complexes with low coordination numbers (< 6) tend to exhibit lone pair activity and hemidirected geometries, while high coordination numbers (> 8) often suppress lone pair influence, leading to holodirected, more symmetrical structures 66-69. Larger ligands or those that promote higher coordination numbers generally reduce lone pair activity, contributing to increased stability and symmetry. The industry standard chelator TCMC does achieve superior complex symmetry and kinetic inertness *in vivo*, by having a high coordination number of 8 which provides, by being bulky and flexible, enough donor atoms to achieve quasi-holodirected coordination geometry. By achieving this, the lone pair no longer dictates geometry or influences stability *in vivo* 66.

However, this is a general trend, and both steric hindrance and the electronic properties of the ligands also play a critical role in modulating the lone pair behavior of Pb²⁺, including in lead-212 complexes 34,64,65,70,71.

## Bifunctional Chelating Ligands for Lead and Practical Considerations

Bifunctional chelators play a pivotal role in the development of novel radiopharmaceuticals, particularly those involving radiometals 13. They are designed with two distinct functional domains: the first forms a stable coordination complex with the radiometal, ensuring *in vivo* stability by preventing transchelation or transmetalation; the second enables covalent attachment to a vector molecule. Common strategies for this linkage include the formation of amide or thiourea bonds, with amide bonds generally offering superior stability, including enhanced resistance to radiolysis. The ideal bifunctional chelator-radionuclide complex should be both thermodynamically stable and kinetically inert, and the complexation should take place under mild radiolabeling conditions if needed for heat sensitive vectors. The choice of chelator is therefore critical, as it affects not only radiolabeling efficiency, but also the pharmacokinetics, biodistribution and safety profile of the final radiopharmaceutical.

**Figure 7:**
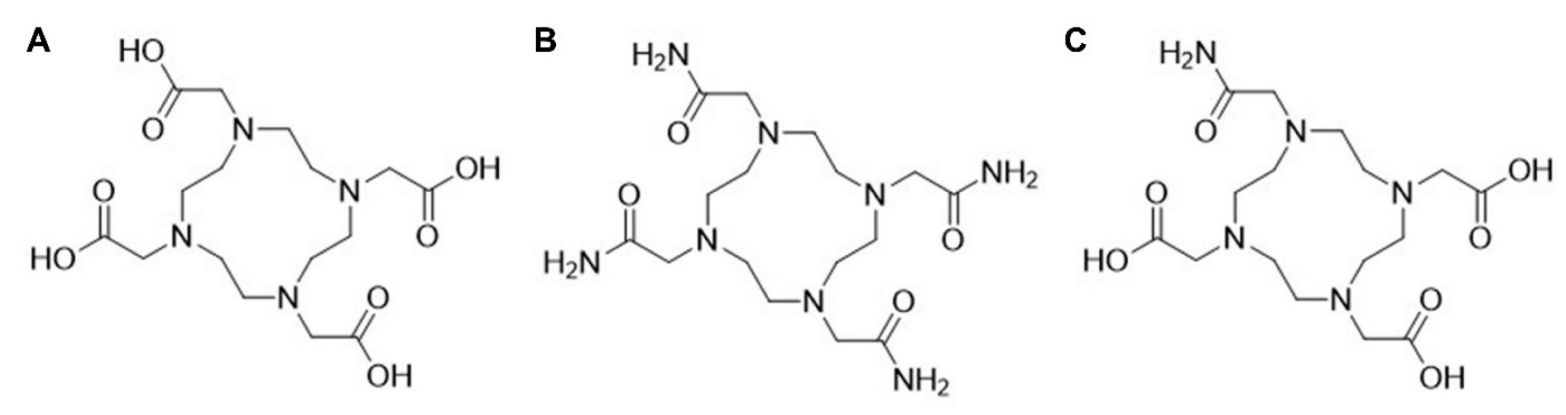
The chemical structures of A) DOTA B) TCMC or DOTAM C) PSC.

Small acyclic ligands such as EDTA and acetylacetonate (ACAC) have proven suboptimal for chelating lead radioisotopes, as they leave coordination sites exposed and do not form sufficiently stable complexes. In contrast, larger macrocyclic ligands like DOTA (1,4,7,10-tetraazacyclododecane-1,4,7,10-tetraacetic acid; Figure 7A) and TCMC (also known as DOTAM, 1,4,7,10-tetraazacyclododecane-1,4,7,10-tetra(2-carbamoylmethyl); Figure 7B) offer improved steric and electronic environments for stable coordination 34. However, the stereochemical behavior of Pb²⁺ within these chelates, specifically whether the 6s² lone pair is holodirected or hemidirected, remains a topic of debate 4,34,72,73.

The combination of a lower coordination number and the asymmetry resulting from the active lone pair often results in weaker bonds and lower stability of the complex. Highly symmetrical complexes with distributed ligand bonds reduce steric strain (crowding of atoms) and this maximizes the strength of the metal-ligand interactions 34. Symmetry also minimizes repulsion between bonded and non-bonded electrons, making the complex thermodynamically more favorable 34,73. In addition to electronic and steric factors, chelation enhances stability by reducing entropy loss upon complex formation. Macrocyclic ligands like DOTA and TCMC pre-organize donor atoms, which leads to improvement of the binding affinity of Pb^2+^ 73.

In addition, to address the unique coordination chemistry of lead, a lead-specific chelator (PSC) has also been developed, designed to optimize complexation specifically for lead radioisotopes 55.

### DOTA and TCMC

Chelators that incorporate hard donor atoms, such as the oxygen atoms in DOTA, contribute significantly to complex stability through strong metal-ligand interactions 34. However, soft donor atoms, typically offer even greater binding affinity for soft (Lewis) acids like Pb²⁺, especially in low-coordination environments where lone pair effects are more pronounced 74,75.

In practice, when compared to DOTA, the nitrogen groups in TCMC provide softer donor capacity and are more favorable for a borderline acid like Pb^2+^ The soft-soft interactions between Pb^2+^ and the TCMC ligand are stronger and more stable than that of the hard-soft interactions between Pb^2+^ and oxygen present in DOTA 55,73,74.

High coordination ligands, such as TCMC, mitigate lone pair effects and ensure symmetrical complexation, leading to greater overall stability 34. Although DOTA provides a rigid macrocyclic framework, its coordination with Pb^2+^ does not fully suppress lone pair activity, whereas TCMC achieves higher symmetry and stability (empirically proven) with less effect of the lone pair on geometry 73,74,76.

### PSC

The PSC chelator (1,4,7,10-tetraazacyclododecane-1,4,10-triacetic acid, 7-(2-carbamoylmethyl)), incorporates a strategic mix of amide and carboxylic acid donor groups. This balance enables PSC to match the electronic preferences of Pb^2+^, optimizing both affinity and kinetic stability 55. In addition, PSC demonstrates a zero formal charge when coupled to lead, which is in contrast with [Pb]DOTA and [Pb]TCMC, demonstrating a net formal charge of -2 in the case of DOTA in its fully deprotonated form, and +2 in the case of TCMC 55. It is postulated that this neutral charge might lead to additional stability and retainment of the bismuth-212 daughter using the PSC chelator 55. Ligands with nitrogen donors enhance lone pair activity through covalent interactions, reducing the stability of the complex, but if combined with oxygen-pendent arms (e.g., PSC), this can be balanced out. Interestingly a recent study by Saidi and co-workers 77 demonstrated that between DOTAM, PSC or DOTA, DOTAM still demonstrated superior chelation efficacy and faster kinetics paired with less kidney accumulation. We expect more information on the ideal chelation strategy for lead-212 to become available.

### Other chelators

Novel chelator development for lead-212 is an active field, and more stable complexes have been reported. For example, Tosato *et al.* report the exploration of several macrocyclic ligands of which DO2A2S and DO3SAm, both DOTA derivatives with varying pendant arm softness, demonstrated the highest complexation efficiency. Additionally, chelators with different macrocyclic backbones, TACD3S, TRI4S, and TE4S were explored but showed no superiority 78. Macropa, a macrocyclic chelator with two pendant picolinic side arms, was studied for radiolabeling of lead-212 by Blei *et al.* 61, 79. The acyclic DTPAm chelator showed promise for chelating lead-203 in one study although no measurement of kinetic stability was reported 80. It was not our aim to provide an exhaustive review of all the novel chelators that are currently under investigation, but we refer the reader to the recent review by Scaffidi-Muta and Abdell for a discussion (including structures) of standard chelators, acyclic chelators, cyclen macrocycles and alternative macrocycles that have been applied for chelation of lead isotopes 13.

### Recoil and Coordination Chemistry Challenges

A persistent challenge in the development of lead-212-based radiopharmaceuticals is the potential decoupling of the daughter nuclide bismuth-212 from the chelator during decay. This can occur due to two key mechanisms: (1) the physical recoil energy released during beta decay, which may disrupt the radiometal-chelate complex, and (2) subtle changes in coordination chemistry between the parent lead-212 and the daughter bismuth-212, leading to reduced binding affinity. It is however commonly accepted that the recoil energy (mechanism 1) is insufficient to break metal ligand bonds, and that the reorganization of valence electrons (mechanism 2) is mostly to blame for the release of the bismuth daughter upon decay 29. Upon beta-minus decay, Pb^2+^ becomes Bi^3+^, and bismuth has a smaller ionic radius compared to lead and is a harder Lewis acid which prefers oxygen donors.

In contrast to some other alpha emitters, the choice of chelator seems to have at least some mild influence on the stable chelation of lead-212 and progenies 81. Specifically, the recoil effect following the beta-minus decay of lead-212, with typical recoil energies in the order of a few eV, is much less pronounced than that observed in alpha emitters such as actinium-225, where alpha decay recoil energies can exceed 100 keV 82.

For example, a study investigating the chemical transformation of [^212^Pb]Pb-DOTA to [^212^Bi]Bi-DOTA reported that approximately 36 ± 2% of the resulting bismuth-212 was unchelated 29. This issue is of particular concern due to the known renal accumulation and toxicity associated with free bismuth-212. Supporting this, [^203^Pb]Pb-DOTA-biotin was found to be stable for up to four days, whereas [^212^Pb]Pb-DOTA-biotin showed over 30% release of bismuth-212 after just four hours 83. Although the data are unpublished, Maaland *et al.* reported that the use of TCMC as a chelator reduced bismuth-212 release to approximately 16% 84, suggesting an improvement in daughter retention. Conflicting results were presented by Westrøm and co-workers suggesting the value to be closer to 30%, similar to that reported for [Pb]DOTA 14. If chelator decoupling happens *in vivo*, this effect will not be depicted by lead-203 based imaging, as this radioisotope does not have bismuth-212 in its decay scheme.

Despite ongoing research into alternative chelators, TCMC remains one of the most widely used bifunctional chelators for lead-based radiopharmaceuticals. Its strong coordination with Pb²⁺ ions, excellent *in vivo* stability, and commercial availability make it a reliable choice for both clinical and investigational use. Clinically relevant examples include [²¹²Pb]Pb-TCMC-trastuzumab for HER2-positive cancers and [²¹²Pb]Pb-DOTAM-TATE, targeting somatostatin receptors in neuroendocrine tumors. TCMC's established safety profile and performance continue to underpin its dominance in the field of lead-based TAT.

## Preclinical and Clinical Proof-of-concept Studies with Lead-based Radiopharmaceuticals

**Figure 8:**
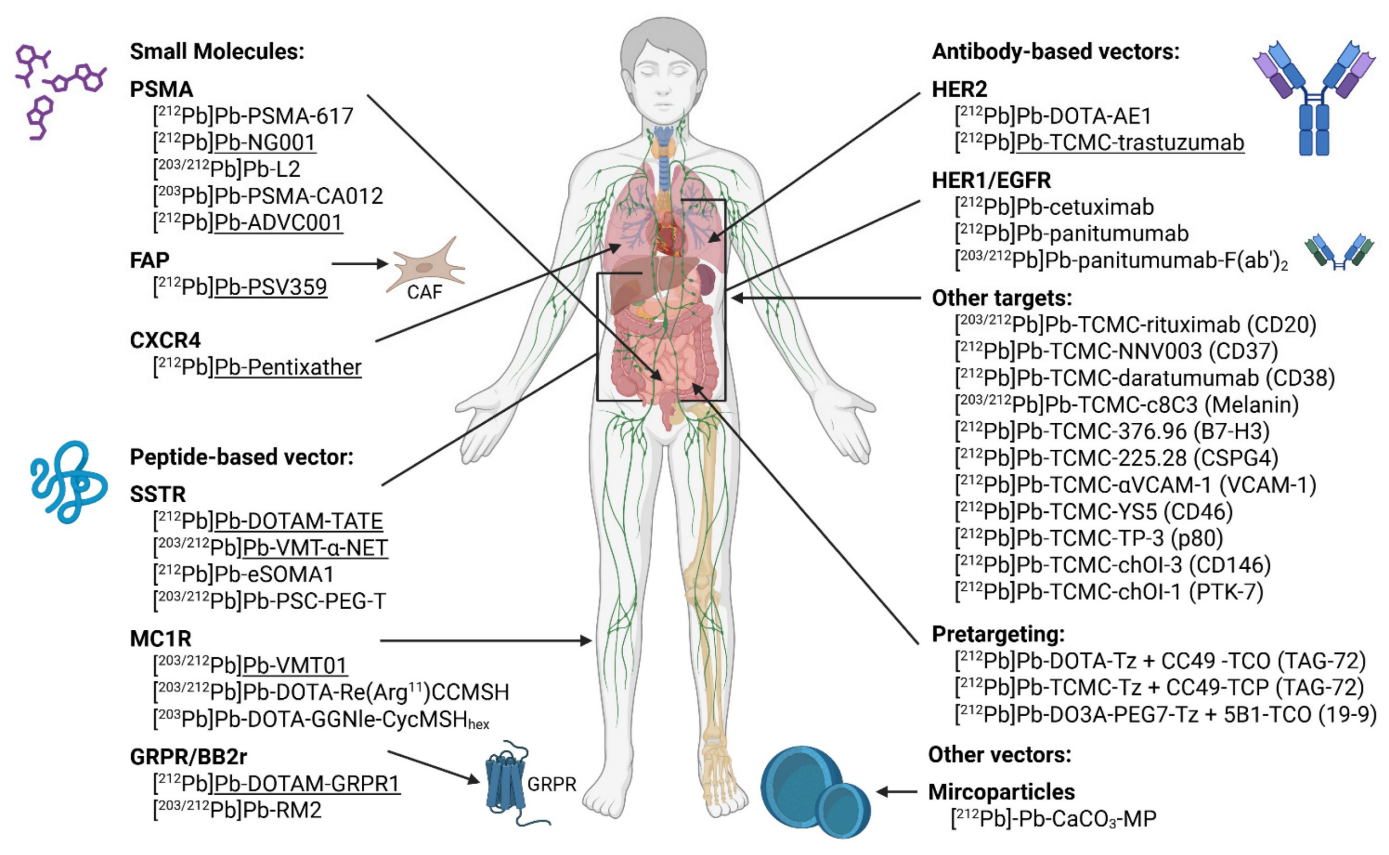
Potential targets investigated in preclinical and clinical studies using lead-212 or lead-203 based radiopharmaceuticals. Classified based on type of vector molecule and target. Constructs being evaluated in clinical trials are underscored. PSMA: prostate-specific membrane antigen; FAP: fibroblast activating protein; CXCR4: C-X-C motif chemokine receptor 4; SSTR: somatostatin receptor; MC1R: melanocortin-1 receptor; GRPR; gastrin-releasing peptide receptor; BB2r: bombesin subtype 2 receptor; HER2/1: human epidermal growth factor receptor 2/1; EGFR: epidermal growth factor receptor; VCAM: vascular cell adhesion molecule; PTK-7: protein tyrosine kinase 7; MP: microparticles.

Given the limited understanding of the stability, radiobiology, and therapeutic efficacy of ^212^Pb-based radiopharmaceuticals, ongoing research is essential to assess their feasibility for clinical application. A summary of currently investigated targets and radiopharmaceuticals (both preclinical and clinical) is summarized in Figure 8. Drawn with a licensed version of BioRender.com.

The choice of vector molecule paired with lead radioisotopes has profound impact on the biological behavior of the radiolabeled compound. Given the relatively short half-life of lead-212, small molecules and peptides provide the best matched pharmacokinetic profile with a similar biological half-life 53. They have the advantage of a rapid tissue penetration, distribution and relatively fast clearance from the systemic circulation 85.

In contrast, full-length antibodies have more limited tumor penetration and a prolonged systemic circulation, which is not ideal given the half-life of lead-212. However, antibodies are still among the most widely investigated vectors in TAT due to their high target specificity 85. Moreover, this mismatch in biological and radiopharmaceutical half-lives can be mitigated in certain cases by compartmental administration routes, such as intraperitoneal (i.p.) injection, particularly for targeting disseminated peritoneal disease 86. Antibody fragments offer a promising alternative, retaining target specificity while exhibiting improved tumor penetration and faster clearance, making them more compatible with the pharmacokinetics of lead-212 87.

Lastly, nanotechnology such as microparticles have also been explored for lead-212-based TAT. These systems can be engineered for multivalent targeting and enhanced tumor retention but pose additional challenges related to biodistribution and clearance 88. This review provides an overview on all lead-212-based constructs evaluated in preclinical models, categorized by their vector type and target, with reference to their lead-203 theranostic counterpart where applicable. Moreover, a list of clinical trials registered on clinicaltrials.gov (accessed 2025/09/01) is provided in Table 2.

### Small molecule and peptidomimetic-based radiopharmaceuticals

#### Prostate-specific membrane antigen (PSMA)

PSMA-targeted small molecules have been widely applied clinically for RNT, especially labeled with lutetium-177 but also alpha emitters like actinium-225 89. While PSMA is limitedly expressed in healthy tissues, a majority of prostate cancer metastases are found to overexpress PSMA, making PSMA an ideal target for TRT 90​.

##### Preclinical research

**Figure 9:**
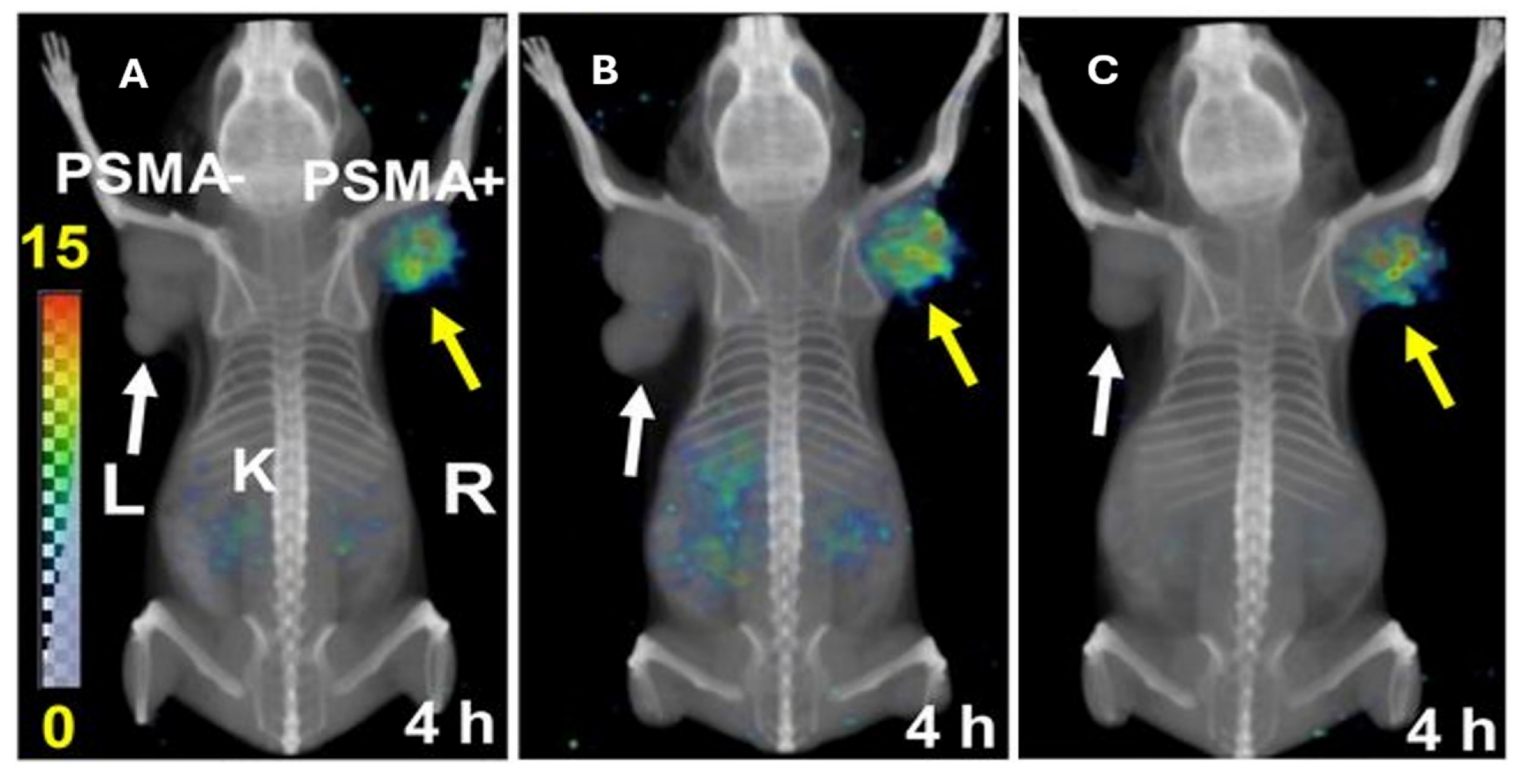
Whole-body volume rendered SPECT in PSMA+ (PC3 pip) and PSMA- (PC3 flu) tumors. The distribution of A) [^212^Pb]Pb-L2, B) [^212^Pb]Pb-L3 and C) [^212^Pb]Pb-L4 at 4 hours are depicted, with [^212^Pb]Pb-L2 showing the most optimal biodistribution for further evaluation (reprinted with permission from JNM. Banerjee et al., 2020,61:80-88. 93).

Compared to the reference DOTA conjugated [^212^Pb]Pb-PSMA-617 construct, [^212^Pb]Pb-NG001, a TCMC-conjugated PSMA ligand, demonstrated comparable tumor uptake and reduced kidney retention when evaluated in C4-2 (PSMA-expressing prostate cancer cell line) bearing mice 90,91​. In a follow-up study, [^212^Pb]Pb-NG001 treatment demonstrated an activity-dependent delay in tumor growth and prolonged survival in human prostate cancer (PC-3 PIP) bearing mice. Fractionated administration seemed to enhance this beneficial result 92. Many ^212^Pb-labeled PSMA ligands have been described in the literature. A series of low-molecular-weight PSMA ligands (L1-L5) were developed based on the 4-bromobenzyl derivative of lysine-urea-glutamate and subsequently evaluated preclinically after radiolabeling with both lead-203 and lead-212 (Figure 9). All the TCMC-based agents demonstrated lower renal retention compared to DOTA-based variants. A subsequent* in vivo* therapy study showed [^212^Pb]Pb-L2 delayed tumor growth successfully in PSMA-positive PC3 PIP xenografts and micro-metastatic models ​^93^. Dos Santos *et al.* introduced another PSMA ligand for lead-203/lead-212 theranostic use. [^203^Pb]Pb-PSMA-CA012 presented with optimal uptake, and a first-in-human dosimetry study was subsequently performed using [^203^Pb]Pb-PSMA-CA012 in two prostate cancer patients. However, this study only reported a theoretical extrapolation to [^212^Pb]Pb-PSMA-CA012 without actual preclinical assessment 94.

##### Clinical research

Patients with progressive, PSMA-positive metastatic castration-resistant prostate cancer (mCRPC) after androgen receptor pathway inhibitors and chemotherapy are eligible for the EMA- and FDA-approved therapy [^177^Lu]Lu-PSMA-617 (Pluvicto) 95. However, up to 30% of these patients either fail to respond or develop resistance to this beta-RNT 96. In such cases, PSMA ligands labeled with alpha emitters may provide improved therapeutic efficacy. An early phase 1 trial (NCT05725070) investigated the imaging potential of [^212^Pb]Pb-NG001, a PSMA-targeted radioligand, in three patients with progressive mCRPC selected by [^18^F]PSMA-1007 PET imaging. Each subject received a single intravenous (i.v.) micro-amount of [^212^Pb]Pb-NG001 (9.4 ± 0.3 MBq). Planar and SPECT/CT gamma camera imaging was performed 1-3 hours and 16-24 hours after radioligand injection. Blood analyses confirmed the post-injection stability of the radioligand. Although gamma camera imaging was feasible, the low injected activity presented challenges. Nonetheless, [^212^Pb]Pb-NG001 effectively targeted mCRPC, demonstrated a favorable biodistribution profile with potentially low uptake in the salivary glands, and was well-tolerated without adverse effects at these subtherapeutic micro-amounts 97.

**Figure 10:**
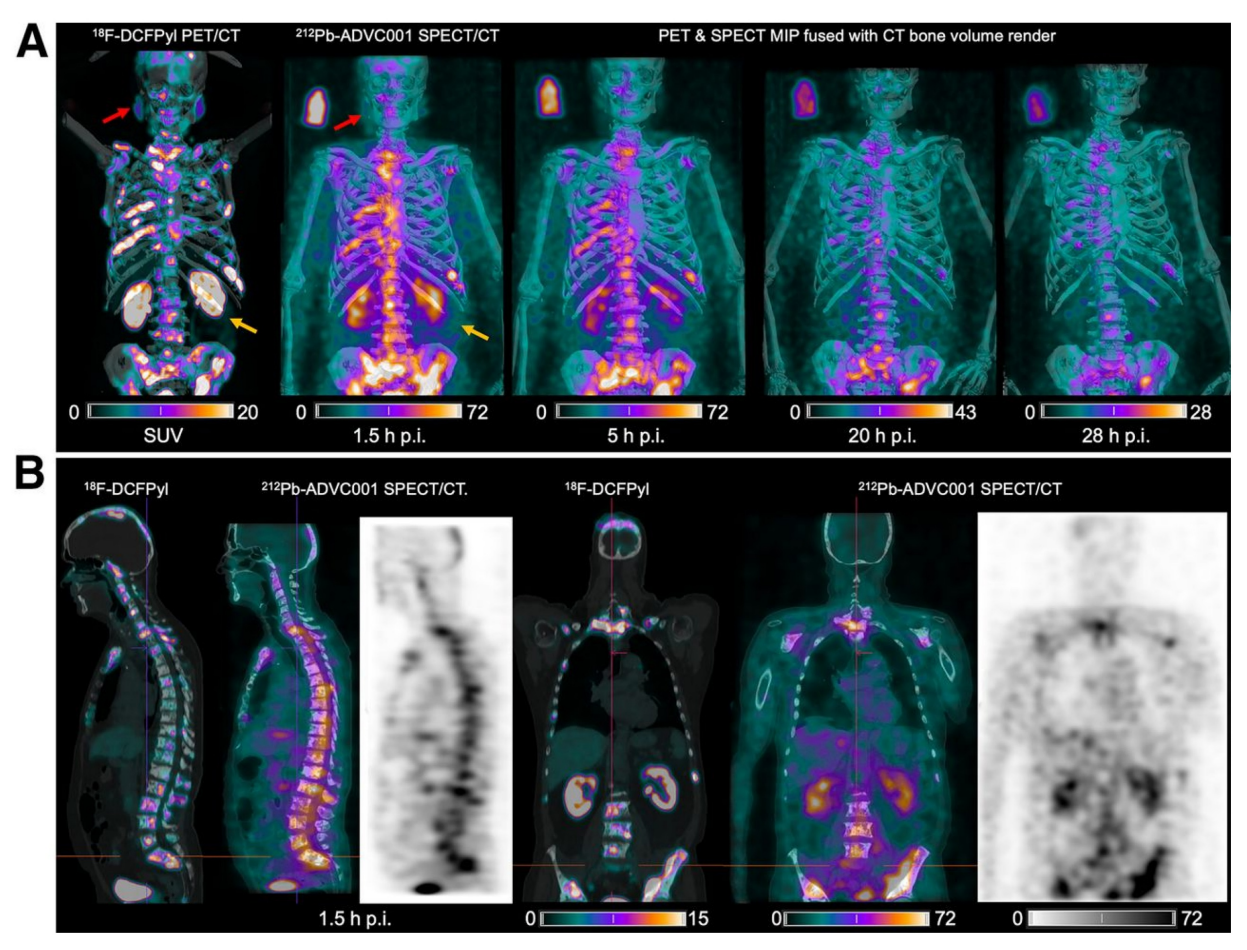
A) PET/CT images taken with [^18^F]DCFPyl concurrently with SPECT/CT images of [^212^Pb]Pb-ADVC001 demonstrating superior dosimetry for salivary glands and kidney clearance for the lead-212 therapeutic. B) Sagittal and coronal images are presented after a 1.5-hour biodistribution time. This research was originally published in *JNM*. Griffiths et al. First-in-human ^212^Pb-PSMA-Targeted α-therapy SPECT/CT imaging in a patient with metastatic castration-resistant prostate cancer. J Nucl Med. 2024;65(4);664. © SNMMI 98.

Another PSMA-targeting agent, [^212^Pb]Pb-ADVC001 (Figure 10) is under evaluation in a phase 1/2 trial (NCT05720130). In this study, up to 18 patients will receive the radioligand in a maximum of four cycles, following a 3+3 dose escalation design. Cohort administered activities include 60 MBq, 120 MBq, 160 MBq, and 200 MBq of [^212^Pb]Pb-ADVC001. Once the recommended phase 2 activity is determined, the study will advance to an expansion phase with three patient groups. One patient in the trial, a 73-year-old man, received 60 MBq of [^212^Pb]Pb-ADVC001 and underwent SPECT/CT imaging (photopeaks at 78 keV and 239 keV) at 1.5, 2, 20, and 28 h post-injection. Summed images reconstructed from both energy windows showed tumor biodistribution consistent with pretreatment [^18^F]F-DCFPyl PET/CT scans, together with low salivary gland uptake and rapid kidney clearance. These findings further demonstrate the feasibility and utility of lead-212 SPECT/CT imaging for assessing radiopharmaceutical biodistribution and enabling patient-specific dosimetry in the clinical development of lead-212-based TAT 98.

#### Fibroblast activated protein (FAP)

Another promising target gaining interest is fibroblast activation protein (FAP), predominantly expressed on cancer-associated fibroblasts (CAFs) within the tumor microenvironment. CAFs are known to promote tumor growth and metastasis, stimulate angiogenesis and suppress the immune response. Therefore, targeting CAFs may provide substantial therapeutic benefit beyond the eradication of neighboring tumor cells. Early therapeutic approaches have focused on lutetium-177-labeled FAP ligands 99. However, lead-212 may be a more suitable option due to its shorter half-life, which better aligns with the relatively brief tumor retention time of early generation FAP ligands. Strategies to enhance tumor retention, such as multivalent FAP constructs and covalent binding approaches, are currently under investigation.

##### Clinical research

An upcoming clinical trial (NCT06710756) will evaluate the imaging and therapeutic potential of a lead-203 or lead-212-labeled cyclic peptide, PSV359, in patients with FAP-expressing solid tumors. However, no results have been reported so far.

#### C-X-C motif chemokine receptor 4 (CXCR4)

Activation of the C-X-C motif chemokine receptor 4 (CXCR4) by its ligand, C-X-C motif chemokine ligand 12 (CXCL12), drives tumor growth and metastasis 100. Targeting the CXCR4/CXCL12 axis with [^177^Lu]Lu-Pentixather, a lutetium-177-labeled CXCL12-analogue, has demonstrated promising therapeutic efficacy in a limited group of patients with hematological malignancies, including multiple myeloma and lymphomas 101.

##### Clinical research

A lead-212-labeled version of Pentixather will be investigated in patients with atypical lung carcinoids, with two infusions administered six weeks apart (NCT05557708). To guide patient selection and assess treatment response, SPECT imaging with [^203^Pb]Pb-Pentixather will be performed before treatment and three months post-treatment.

### Peptide-based radiopharmaceuticals

#### Somatostatin receptor (SSTR)

The treatment of SSTR-expressing neuroendocrine tumors (NETs) was significantly enhanced with the development of [^177^Lu]Lu-DOTATATE (Lutathera), which was the first EMA- and FDA-approved lutetium-177 based radiopharmaceutical. Based on this effective application of somatostatin analogues for peptide receptor targeting radiotherapy (PRRT) 102​, investigations to optimize PRRT using alpha emitters, were initiated, including lead-212-based PRRT 103.

##### Preclinical research

Preclinical investigations demonstrated [^212^Pb]Pb-DOTAMTATE, in which the TATE vector peptide (Tyr^3^-octreotate) is functionalized with TCMC as a chelator, to have a high tumor uptake in AR42J pancreatic tumor immunocompromised xenografts. Uptake in the pancreas and kidneys was shown to be a limiting factor, but it could be mitigated by nephroprotective strategies. Interestingly, a combination with the chemotherapeutic agent 5-fluorouracil, a well-known radiosensitizer, showed promising efficacy in this study 104. Further optimization includes novel vector molecules like eSOMA-01, which demonstrated an optimal tumor-to-kidney uptake ratio (Figure 10) 56, and the incorporation of the PSC chelator via a PEG_2_ linker in [^212^Pb]Pb-PSC-PEG-T (later renamed as VMT-α-NET), showing good tumor accumulation and retention with fast renal clearance. Moreover, this study highlighted the theranostic potential of the lead-203/lead-212 pair through SPECT imaging of [^203^Pb]Pb-VMT-α-NET confirming the biodistribution profile 55​. It has to be noted that not only did these compounds differ in their chelating strategies but also utilized different targeting peptides (octreotate vs octreotide).

When radiolabeling therapeutic radiopharmaceuticals, optimal molar activities remain an important consideration, and this was also demonstrated by Pretze and co-workers 105 through the measurement of effect of cellular uptake of a SSTR2 targeting [^203/212^Pb]Pb-PSC-PEG2-TOC (later renamed as VMT-α-NET). In this study a higher cellular uptake was proven with a rising molar activity with no limit found in the evaluated window of molar activity. Whilst the broader application of this study on ^212^Pb-radiopharmaceuticals (preclinical and clinical) remains to be determined, we feel it is important to highlight that the influence of molar activity on tumour accumulation should always be considered during the development of novel therapeutic radiopharmaceuticals.

##### Clinical research

**Figure 11:**
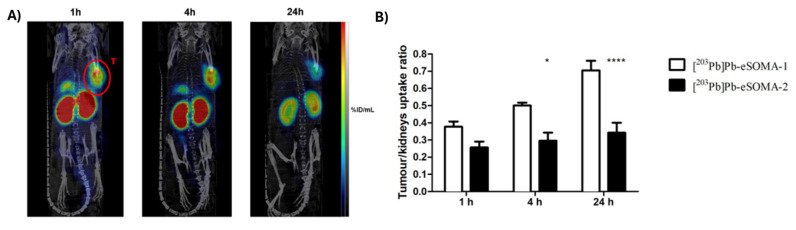
The biodistribution of [^203^Pb]Pb-eSOMA-01 in A) SPECT/CT scans of H69 human small cell lung cancer-bearing mice at different time points up to 24 hours and B) tumor/kidney ratios derived from the SPECT images (Reprinted under the terms and conditions of the Creative Commons Attribution license (CC BY) from 56). Note that the compound [^203^Pb]Pb-eSOMA-2 mentioned in the figure, is a less optimal compound, not discussed.

The first lead-212-labeled SSTR-targeting ligand to reach clinical trials was [^212^Pb]Pb-DOTAMTATE (AlphaMedix^TM^). In a phase 1 injected activity escalation study (NCT03466216), [^212^Pb]Pb-DOTAMTATE was administered i.v. to 20 patients with histologically confirmed NETs expressing SSTR without prior history of RNT 106​. After single ascending amounts of activity with an increment of 30% and a multiple ascending injected activity regimen, the recommended regimen for a phase 2 study was four cycles of i.v. administered 2.5 MBq/kg [^212^Pb]Pb-DOTAMTATE at eight-week intervals. Of the ten subjects receiving the highest amount of activity, eight showed an objective, long-lasting radiologic response according to [^68^Ga]Ga-DOTATATE PET/CT scans (Figure 11). The treatment was well tolerated, with mild adverse events such as nausea, mild hair loss, abdominal pain, diarrhea, and fatigue being observed. These encouraging results led to the ongoing phase 2 study (NCT05153772) in which 66 subjects with or without prior TRT history are included. Preliminary results demonstrate an objective response rate (ORR) of 47.2% 107​. When pooling data from both phase 1 and phase 2 trials, the ORR increases to 50%, surpassing the 18% ORR reported for [^177^Lu]Lu-DOTATATE in the phase 3 NETTER-1 study 102​. The latest results were presented at the European Society for Medical Oncology (ESMO) meeting in October 2025, with separated results for previously treated and PRRT-naïve patients. In 35 PRRT-naïve patients, of whom 86% were able to receive all 4 intended administrations, there was a 60% ORR with an impressive disease control rate (DCR) of 94% and an equally impressive duration of response (DoR) of ≥ 24 months in 72% of the 21 responding patients 108. The PFS rate at 36 months was 73% as assessed by local investigators, the 36 months overall survival (OS) rate was 88%. These findings suggest that targeting SSTR-expressing tumors with alpha-emitting radioligands might outperform the current PRRT therapies utilizing beta-emitters. However, in 17/35 (49%) PRRT-naïve patients dysphagia was described (grade 3 in 1 patient, no grade 4), with an “achalasia-like” presentation and responsiveness to botox injections 107,108. Furthermore, 13/35 (37%) of patients developed a renal event, of which 6 (17%) grade ≥ 3. Further long term follow-up and larger clinical trials will have to demonstrate the safety profile of this radiopharmaceutical in this setting.

In another 26 patients previously treated with PRRT, of whom 84% received all 4 intended therapy administrations, a 35% ORR was observed, with an impressive DCR of 96% and a DoR of ≥18 months in all 9 responding patients 109. The 18-month PFS rate was 83% and the 18-month OS rate was 85%. Dysphagia was again observed, in 15/26 (58%) of patients, of whom 4 (15%) grade 3 (no grade 4). Only 2 of the 26 patients (8%) showed renal adverse events, both grade ≥ 3 108,109. No cases of myelodysplastic syndrome (MDS) or acute myeloid leukemia (AML) were reported in both cohorts (pre-treated or PRRT-naïve) 108,109. Taken together, [^212^Pb]Pb-DOTAMTATE has the potential to become a clinically meaningful therapeutic choice in NET patients, either as first PRRT agent or in PRRT pre-treated patients upon progression.

Another SSTR-targeting ligand under clinical development is VMT-α-NET (previously named PSC-PEG-TOC). A personalized approach has been investigated during clinical evaluation of [^212^Pb]Pb-VMT-α-NET, involving a preliminary dosimetry step with its theranostic partner [^203^Pb]Pb-VMT-α-NET, which is evaluated either in a separate study (NCT05111509) or in an integrated sub-study (NCT05636618). A first case study reported the biodistribution and scintigraphy images of [^212^Pb]Pb-VMT-α-NET in a 75-year-old woman with advanced G2 Net of unknown primary 110. In an early phase 1 trial (NCT05111509), ten patients with beta-PRRT-relapsed or refractory gastro-entero-pancreatic NETs received 185 MBq of [^203^Pb]Pb-VMT-α-NET i.v., followed by SPECT/CT imaging at 1, 4-8, 24-30, and 42-52 hours post-injection. Three participants were also administered nephroprotective amino acids (lysine and arginine) over four hours and scheduled for subsequent treatment with [^212^Pb]Pb-VMT-α-NET as part of a separate phase 1 trial (NCT06148636).

Imaging results from [^203^Pb]Pb-VMT-α-NET were compared to those of gallium-68 [^68^Ga]Ga-DOTA-TATE or [^68^Ga]Ga-DOTA-TOC, and organ-level MIRD dosimetry calculations were used to predict critical organ doses for [^212^Pb]Pb-VMT-α-NET. Predicted organ doses were 14.3 ± 4.6 mGy/MBq for the kidneys, 8.4 ± 4.1 mGy/MBq for the spleen, 2.4 ± 0.8 mGy/MBq for the liver, and 0.59 ± 0.11 mGy/MBq for blood. Based on these calculations, three patients who received nephroprotective amino acids were administered cumulative [^212^Pb]Pb-VMT-α-NET activities of 196.1, 270.1, and 492.1 MBq, respectively, over two cycles to reach the target renal dose of 3.5 Gy for the first cohort of the phase 1 trial (NCT06148636) 111​. Another study reports the investigation of [^203^/^212^Pb]Pb-VMT-α-NET in a larger cohort of patients (12 patients). It was found that imaging with [^203^Pb]Pb- VMT-α-NET followed by a single dose of [^212^Pb]Pb- VMT-α-NET was well tolerated with promising efficacy 112.

In the ongoing integrated phase 1 trial testing the theranostic pair [^212^Pb]Pb-VMT-α-NET and [^203^Pb]Pb-VMT-α-NET (NCT05636618), up to 160 patients with NETs that were not previously treated with TRT will be recruited. In the first two cohorts, subjects received 200 MBq of [^203^Pb]Pb-VMT-α-NET for dosimetry evaluation before [^212^Pb]Pb-VMT-α-NET treatment. SPECT/CT imaging was performed up to 24 hours post-injection, and participants received up to four treatment cycles of [^212^Pb]Pb-VMT-α-NET, co-administered with nephroprotective amino acids, spaced eight weeks apart. Recently, at ESMO 2025, results on 55 treated NET patients have been presented (2 treated with 92.5 MBq/cycle, 46 with 185 MBq/cycle and 7 with 222 MBq/cycle) 113. The most common treatment-emergent adverse event was lymphocytopenia, observed in 33% of patients (minority grade 3, no grade 4). No activity limiting toxicities nor grade 4/5 adverse events were reported. Interestingly, there were no serious renal complications and no dysphagia. Objective partial response was seen in 8/23 (35%) evaluable patients in cohort 2 (185 MBq/cycle) with ≥ 9 months follow-up, with stable disease in 14/23 (61%) and one patient with a non-interpretable scan. The ORR increased to 44% in patients with SSTR2 positivity in all tumoral lesions (n = 16). The 2 patients treated with 92.5 MBq/cycle still had stable disease ≥ 18 months after treatment 113. Further clinical investigation of this clearly promising radiopharmaceutical is ongoing. Furthermore, two additional clinical trials (NCT06479811 and NCT06427798) investigating [^212^Pb]Pb-VMT-α-NET in patients with SSTR-expressing tumors are expected to start recruitment in 2025.

#### Melanocortin-1 receptor (MC1R)

Melanotropin peptide analogues targeting the melanocortin-1 receptor (MC1R), which is overexpressed in melanoma cells, have been explored as vector molecules for lead-203/lead-212 theranostics 114.

##### Preclinical research

**Figure 12:**
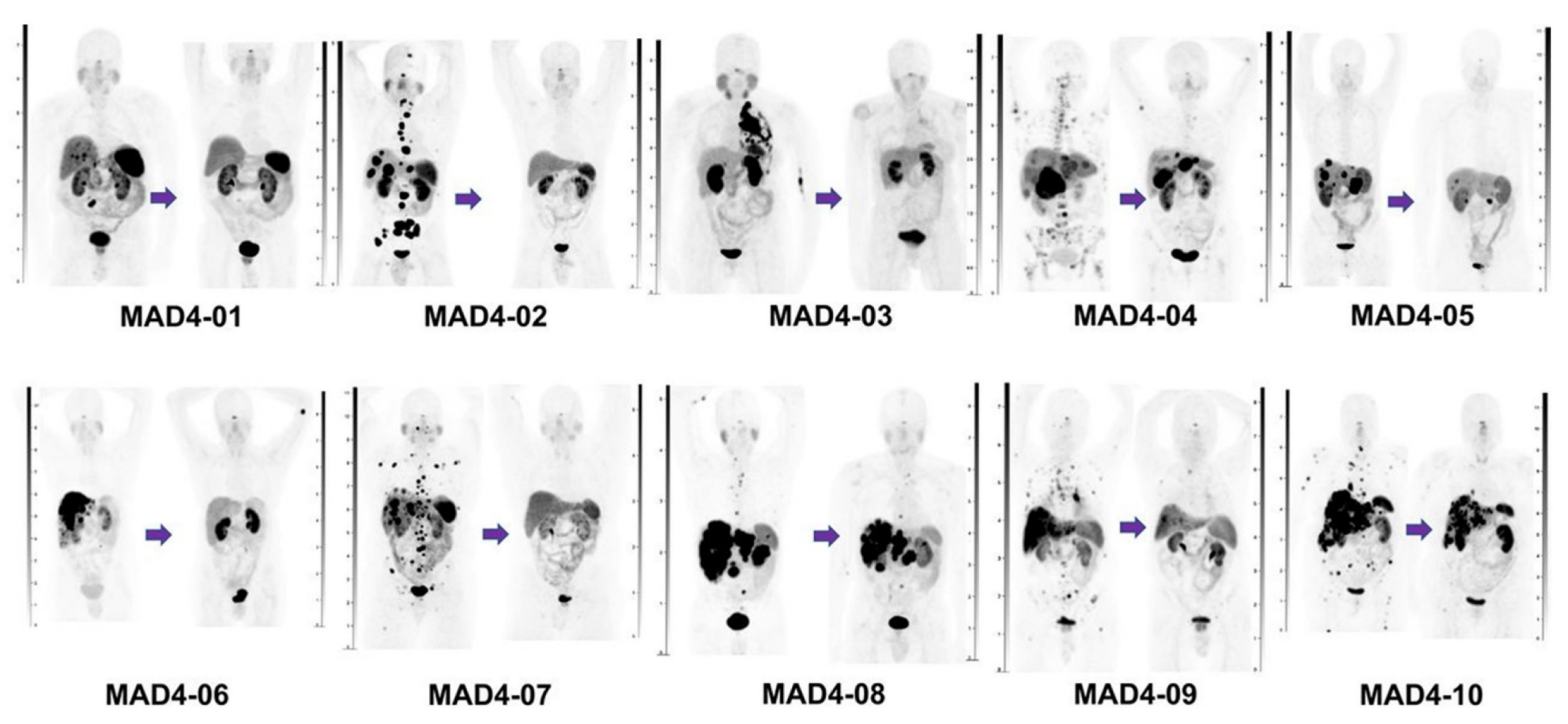
Maximum intensity projection (MIP) images of [^68^Ga]Ga-DOTATATE PET/CT images of the first ten subjects before treatment (left scan of each patient) and after receiving four cycles of [^212^Pb]Pb-DOTAMTATE therapy (right scan of each patient) at an injected activity level of 2.5 MBq/kg for each cycle [Reprinted with permission from JNM, Delpassand et al., 2022;63:1326-1333. [106]].

The radiolabeled peptide targeting MC1R, [^212^Pb]Pb-DOTA-Re(Arg^11^)CCMSH, demonstrated rapid tumor uptake, prolonged tumor retention, and injected activity-dependent survival benefit in B16/F1 melanoma-bearing immunocompetent xenografts 114​. The lead-203 counterpart, [^203^Pb]Pb-DOTA-Re(Arg^11^)CCMSH (Figure 12) confirmed the promising pharmacokinetics and suitability as a theranostic pair 115​. Another MC1R targeting peptide, [^203^Pb]Pb-DOTA-GGNle-CycMSH_hex_ demonstrated a favorable accumulation and good imaging properties in both immunocompetent subcutaneous B16/F1 and B16/F10 mouse models, as well as in a B16/F10 pulmonary metastatic melanoma mouse model 116​. Lastly, [^212^Pb]Pb-VMT01, also targeting the MC1R receptor, showed to reduce tumor growth in B16/F10 melanoma-bearing immunocompetent mice. Biodistribution of the [^203^Pb]Pb-VMT01 theranostic counterpart showed rapid tumor accumulation with off-target accumulation primarily localized in the kidneys. Interestingly, based on the induced immunological cell death by TAT, this study reported additional tumor cytotoxicity when combining [^212^Pb]Pb-VMT01 administration with immune checkpoint inhibitor therapy 117​.

##### Clinical research

A theranostic approach involving lead-based radioligands was evaluated for MC1R-expressing metastatic melanoma patients. This strategy employed the theranostic pair [^203^Pb]Pb-VMT01 for patient selection and dosimetry assessment and [^212^Pb]Pb-VMT01 for TAT. The imaging TIMAR1 trial (NCT04904120) used a cross-over design in stage IV melanoma patients, who received randomized, sequential i.v. injections of activities of 555-925 MBq [^203^Pb]Pb-VMT01, followed by SPECT/CT imaging at 1, 4, and 24 h post-injection, and 74-277 MBq [^68^Ga]Ga-VMT02, with PET/CT imaging performed dynamically up to one hour and at two and three hours post-injection, approximately one month apart. Only three subjects underwent imaging with [^203^Pb]Pb-VMT01.

The experimental tracer [^68^Ga]Ga-VMT02 successfully identified MC1R-positive tumors in three out of six subjects, facilitating patient selection for future [^212^Pb]Pb-VMT01 therapy trials 118. SPECT/CT imaging demonstrated tumor retention of [^203^Pb]Pb-VMT01 at 24 h, supporting organ and tumor dosimetry calculations applicable to [^212^Pb]Pb-VMT01 treatments. Subsequently, a phase 1/2 study (NCT05655312) evaluating [^212^Pb]Pb-VMT01, incorporated positive [^203^Pb]Pb-VMT01 or [^68^Ga]Ga-VMT02 imaging as an inclusion criterion 119. This injected activity escalation and injected activity expansion study plans to enroll up to 264 subjects, who will receive up to 555 MBq [^212^Pb]Pb-VMT01 i.v., either as monotherapy or combined with the programmed cell death protein 1 (PD-1) inhibitor nivolumab, administered for up to three treatment cycles eight weeks apart. Nephroprotective amino acids will be co-administered. In the initial cohort, patients received 111 MBq of [^212^Pb]Pb-VMT01, while the ongoing second cohort utilizes 185 MBq 119. Recently, the FDA granted fast-track designation to [^212^Pb]Pb-VMT01 in melanoma 120.

#### Gastrin-releasing peptide receptor (GRPR)/bombesin receptor subtype 2 (BB2)

The gastrin-releasing peptide receptor (GRPR), also known as bombesin receptor subtype 2 (BB2), is expressed in various tumors, including prostate, breast, lung, colorectal, cervical cancers, and cutaneous melanoma. Moreover, expression of GRPR/BB2 is limited in healthy tissues, providing a good rationale for GRPR/BB2 targeted RNT 121.

##### Preclinical research

The RM2 peptide targeting the BB2 receptor, expressed in prostate cancer, consists of the structure DOTA-4-amino-1-carboxymethyl-piperidine-D-Phe-Gln-Trp-Ala-Val-Gly-His-Sta-Leu-NH_2_. This peptide has been radiolabeled with both lead-203 and lead-212 for preclinical evaluation. [^203^Pb]Pb-RM2 was shown to exhibit long-term tumor retention and great complex stability up to 24 hours post injection in PC-3 immunocompromised xenografts 122. Tumor targeting was confirmed following administration with the therapeutical radiopharmaceutical [^212^Pb]Pb-RM2. Furthermore, treatment of immunocompromised prostate cancer (PC-3)-bearing mice indicated good tolerability and temporary control of tumor growth 123.

##### Clinical research

A ^212^Pb-labeled GRPR antagonist, [^212^Pb]Pb-DOTAM-GRPR1, is currently under investigation in a phase 1 study (NCT05283330) for subjects with recurrent or metastatic GRPR-expressing tumors, who have not previously received PRRT. The injected activity escalation phase follows a 3+3 design, with activity increments of approximately 30% in subsequent cohorts. The maximum allowable total injected activity per cycle is 203.5 MBq. In the multiple-ascending injected activity regimen, participants may receive up to 888 MBq over four cycles. Once the recommended multiple-ascending activity is determined, patients will receive up to four cycles of [^212^Pb]Pb-DOTAM-GRPR1 at eight-week intervals. No results have been published to date.

### Antibody-based radiopharmaceuticals

#### Human epidermal growth factor receptor 2 (HER2)

Antibodies have been extensively studied as targeting vectors for lead-212 based TAT in preclinical research, with most research focusing on human epidermal growth factor receptor 2 (HER2) targeting constructs. HER2 is found to be overexpressed in approximately 20-30% of breast cancers, as well as a substantial proportion of ovarian, gastric and lung adenocarcinomas, and can be correlated with poor prognosis in these patients 124.

##### Preclinical research

Early work by Horak *et al.* 125 evaluated [^212^Pb]Pb-DOTA-AE1 in murine SK-OV-3 ovarian cancer-bearing immunocompromised mice. Herein, the AE1 targeting vector consisted of an IgG2a murine monoclonal antibody targeting the HER2/neu receptor. Treatment with [^212^Pb]Pb-DOTA-AE1 was shown to halt the development of small tumor growth but was ineffective in larger tumors 125. A major limitation of this study is the potential release of bismuth-212 from the DOTA complex during lead-212 decay (36%), resulting in increased renal toxicity, as well as high bone marrow suppression after i.v. injection due to long blood half-life 125.

Milenic *et al.* successfully radiolabeled trastuzumab (Herceptin), an FDA and EMA-approved humanized monoclonal antibody, with lead-212 using TCMC. Therapeutic efficacy was demonstrated in immunocompromised LS-147T colon and Shaw pancreatic carcinoma models 126. Follow-up studies in LS-174T-bearing immunosuppressed mice revealed that [^212^Pb]Pb-TCMC-trastuzumab increased double-stranded DNA breaks, leading to DNA repair inhibition, G2-M cell cycle arrest, and chromatin remodeling 127. The same group later demonstrated that combination therapies with [^212^Pb]Pb-TCMC-trastuzumab enhance therapeutic efficacy. In immunocompromised preclinical models, pairing [^212^Pb]Pb-TCMC-trastuzumab with gemcitabine increased survival and apoptosis in S-phase arrested cells 128,129. Paclitaxel pretreatment improved survival 3.9-fold 130, while pretreatment with carboplatin enhanced median survival up to 7.9-fold. However, combination response was highly dependent on the administration timing in LS-174T -bearing immunocompromised mice 131.

Another group further evaluated [^212^Pb]Pb-TCMC-trastuzumab as a treatment for prostate cancer. Herein, i.v. administration resulted in decreased tumor growth in mice bearing human PC-3MM2 prostate tumors, which express HER2 in low levels. Prolonged survival with no toxicity was also reported 132. The biodistribution and toxicity of [^212^Pb]Pb-TCMC-trastuzumab was analyzed in nonhuman primates, 8 hours, 10 days or 90 days post administration. Up to 48 hours after i.p. injection of the treatment, 95.5 ± 5.0% of activity was retained in the peritoneal cavity as determined by quantitative gamma camera imaging, with no treatment-related toxicity observed 133. These preclinical data on [^212^Pb]Pb-TCMC-trastuzumab have supported further clinical trials.

##### Clinical research

The first phase 1 clinical trial (NCT01384253) involving the use of lead-212 as the cargo for TAT was conducted in 2014, employing [^212^Pb]Pb-TCMC-trastuzumab in patients with HER2 expressing peritoneal carcinomatosis. These patients received 4 mg/kg trastuzumab i.v., followed by a single i.p. injection of 7.4 MBq/m^2^ [^212^Pb]Pb-TCMC-trastuzumab to evaluate the conjugate's distribution, pharmacokinetics, and safety profile. Whole-body gamma scans indicated minimal redistribution out of the peritoneal cavity and minimal uptake in normal organs 134. Subsequently, an activity-escalation trial was initiated, involving 18 patients (16 females with recurrent HER-2 positive ovarian cancer and two males with HER2-positive colon cancer), who were administered escalating activities of 7.4 to 27.4 MBq/m^2^ with a 30% increase for each subsequent activity level 135. The ascending administered activities did not result in significant drug-related toxicities, consistent with the dosimetry analysis. At six weeks post-administration, ten of the 18 patients achieved stable disease, while eight out of 18 showed disease progression. Stable disease was notably observed in nine out of 10 patients receiving injected activities of 12.6 MBq/m^2^ or higher. Overall, up to 27.4 MBq/m^2^ of [^212^Pb]Pb-TCMC-trastuzumab administered i.p. was well tolerated, and further activity escalation studies were suggested as a feasible strategy to enhance tumor response 136.

#### HER1/Epidermal growth factor receptor (EGFR)

Besides the HER2 targeting antibody trastuzumab, other antibodies have also been evaluated as vector molecules. For example, two antibodies, cetuximab 137 and panitumumab 138, have been explored preclinically, both targeting the epidermal growth factor receptor (EGFR), also known as HER1, which is found to be overexpressed in several cancers.

##### Preclinical research

In an immunocompromised LS-174T colon carcinoma mouse model, [^212^Pb]Pb-TCMC-cetuximab significantly improved the median survival compared to an ^212^Pb-isotype control. Moreover, pretreatment with gemcitabine, 24 hours before [^212^Pb]Pb-TCMC-cetuximab administration, resulted in further prolongation of the median survival in tumor-bearing mice, while concomitant treatment with carboplatin and [^212^Pb]Pb-TCMC-cetuximab demonstrated lower efficacy. Dual targeting of HER1 and HER2 using [^212^Pb]Pb-TCMC-cetuximab and [^212^Pb]Pb-TCMC-trastuzumab, respectively, showed similar therapeutic benefits in this study 137. [^212^Pb]Pb-TCMC-panitumumab demonstrated superior therapeutic effects in immunocompromised LS-174T tumor xenografts compared to [^212^Pb]Pb-TCMC-cetuximab or the HER2 targeting [^212^Pb]Pb-TCMC-trastuzumab. Prior treatment with either gemcitabine or paclitaxel potentiated the beneficial effect of [^212^Pb]Pb-TCMC-panitumumab alone. The multimodal therapy regime in which topotecan was administered 24 hours prior and 24 hours post [^212^Pb]Pb-TCMC-panitumumab resulted in the greatest therapeutic response in this study 138​.

[^203^Pb]Pb-TCMC-panitumumab-F(ab')_2_, the F(ab')_2_ fragment of the anti-HER1 antibody, panitumumab, showed similar biodistribution and tumor uptake in immunocompromised LS-174T tumor xenograft mice compared to the full-length antibody, [^203^Pb]Pb-TCMC-panitumumab. However, due to their smaller size, F(ab')_2_ fragments have the advantage of faster pharmacokinetics which aligns better with the relatively short half-life of lead-212, and reduced immunogenicity and off target toxicity relating to the lack of the Fc region. Moreover, therapeutic efficacy was similarly proven after treatment of these mice with [^212^Pb]Pb-TCMC-panitumumab-F(ab')_2_. The combination with either gemcitabine or paclitaxel with [^212^Pb]Pb-TCMC-panitumumab-F(ab')_2_ also similarly potentiated the effect on median survival 87​.

#### Other targets

##### Preclinical research

**Figure 13:**
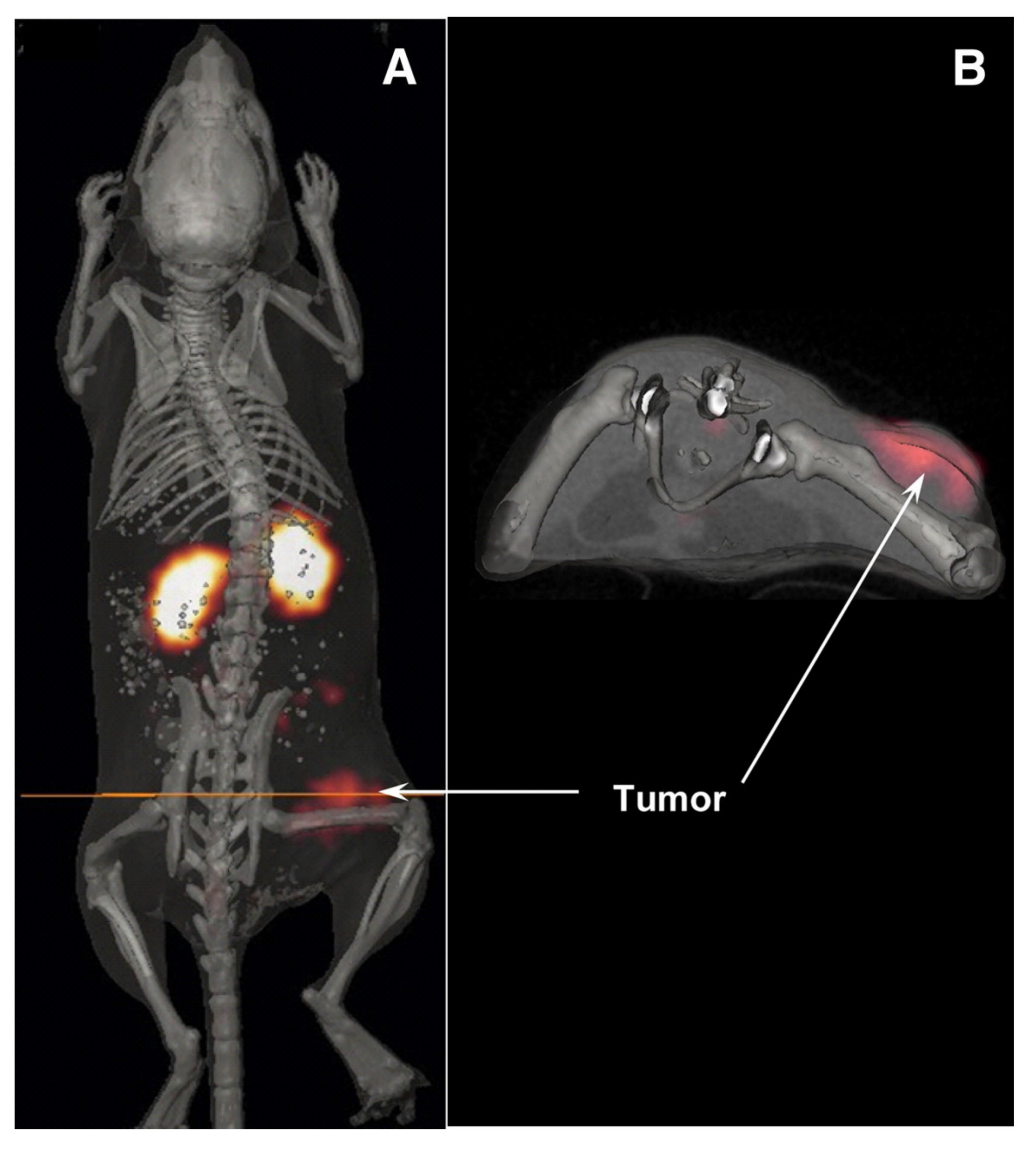
The whole-body (A) and transaxial (B) images of [^203^Pb]Pb-DOTA-Re(Arg^11^)CCMSH 2 hours post-injection in B16/F1 murine melanoma-bearing mice (Reprinted with permission from 115. This research was originally published in JNM. Miao et al. J Nucl Med. 2008;49:823-829).

The lead-203/lead-212 theranostic strategy was applied to CD20-targeting rituximab in non-Hodgkin lymphoma. Tumor retention was demonstrated in immunocompetent EL4-hCD20-Luc bearing mice, but also some off-target uptake in healthy organs. [^212^Pb]Pb-TCMC-rituximab extended survival in both early and late-stage disease 139​. Similarly, for the treatment of non-Hodgkin lymphoma, the CD37-targeting antibody [^212^Pb]Pb-NNV003 showed survival benefits in immunocompromised MEC-2 and Daudi tumor models, though biodistribution revealed rapid uptake in some healthy tissues and slow tumor uptake 84​. One study reported marked tumor growth inhibition in immunosuppressed RPMI8226-bearing mice after [^212^Pb]Pb-TCMC-daratumumab (Figure 13) treatment targeting CD38 in multiple myeloma. However, acute toxicity was reported after i.v. administration at injected activities larger than 277.5 kBq ​^140^.

A theranostic approach using the melanin-targeting antibody c8C3 showed localized tumor uptake of [^203^Pb]Pb-TCMC-c8C3 in immunocompetent B16-F10 melanoma-bearing mice, with the therapeutic analogue [^212^Pb]Pb-TCMC-c8C3 slowing tumor growth in an injected activity-dependent manner. However, high administered activities of the non-targeting control yielded similar benefits in this study 141.

The B7-H3 targeting antibody [^212^Pb]Pb-TCMC-376.96 was evaluated in two immunocompromised ovarian cancer mouse models bearing i.p. ES-2 or A2780cp20 tumors. [^212^Pb]Pb-TCMC-376.96 accumulated in ascites cells and tumors and prolonged survival in an injected activity-dependent manner, while co-treatment with carboplatin showed no added benefit 133​. In pancreatic cancer, administration of [^212^Pb]Pb-TCMC-376.96 in both a patient-derived s.c. Panc039 xenograft model and an orthotopic PDAC3 mouse model resulted in significant tumor uptake and reduced tumor growth in the two immunocompromised models respectively 142.

For triple-negative breast cancer (TNBC), the CSPG4-targeting [^212^Pb]Pb-TCMC-225.28 inhibited tumor growth in immunosuppressed SUM159 xenograft models 143​. Another TNBC therapy, [^212^Pb]Pb-TCMC-αVCAM-1, targeting VCAM-1 in brain metastases, showed a 6-fold higher tumor uptake than healthy brain tissue and significantly reduced metastasis burden compared to standard external beam radiotherapy 51.

In prostate cancer, [^212^Pb]Pb-TCMC-YS5, targeting CD46, inhibited tumor growth and prolonged survival in PC3 and patient-derived xenograft models 144​. For osteosarcoma, [^212^Pb]Pb-TCMC-TP-3, targeting p80, reduced OHS cancer spheroid size *in vitro*, with a proposed dual-alpha strategy combining it with radium-224 145​. In this approach, the bone-seeking radium-224 provides localized irradiation to the osteoblastic matrix, while lead-212, coupled to the osteosarcoma-specific antibody, delivers additional targeted alpha radiation to the tumor cells. In this way, bone tropism and tumor-specific antigen binding are exploited simultaneously to enhance the therapeutic reach of alpha therapy.

I.p. administration of the CD146 targeting murine antibody mOI-3 radiolabeled to lead-212, [^212^Pb]Pb-TCMC-mOI-3, resulted in improved median survival in mice mesothelioma xenografts 146. Similarly, i.p. treatment of [^212^Pb]Pb-TCMC-chOI-1 targeting protein tyrosine kinase 7 (PTK7) was evaluated for the treatment of ovarian cancer, showing tumor retention and uptake into the systemic blood circulation after i.p. injection. Moreover, [^212^Pb]Pb-TCMC-chOI-1 was shown to decrease tumor growth and significantly prolonged survival in two separate immunosuppressed ovarian cancer mouse models 47,147.

#### Pretargeting

##### Preclinical research

Interestingly, pretargeted TAT has been explored in a study by Shah *et al.* in which the administration of the antibody vector was decoupled from the administration of the radionuclide. Utilizing the inverse electron demand Diels-Alder reaction, a tetrazine (Tz) coupled to a chelator, DOTA-Tz or TCMC-Tz, radiolabeled with lead-212 was injected in immunocompromised LS174T bearing mice after pretreatment with the *trans*-cyclooctene (TCO) conjugated CC49 antibody targeting tumor associated glycoprotein 72 (TAG-72). Biodistribution was superior for the [^212^Pb]Pb-DOTA-Tz compared to [^212^Pb]Pb-TCMC-Tz. A reduction in tumor size was seen using the CC49-TCO and [^212^Pb]Pb-DOTA-Tz combination with significantly improved survival *in vivo*, demonstrating the high potential of pretargeted TAT with lead-212 148. A similar study was performed by Bauer *et al.* in which the TCO conjugated antibody 5B1, targeting carbohydrate cell surface antigen 19-9, followed by administration [^212^Pb]Pb-DO3A-PEG_7_-Tz three days later resulted in prolonged survival and delayed tumor growth in immunosuppressed s.c. BxPC-3-bearing mice 149.

### Other vectors

#### Inorganic carrier systems (Microparticles)

Another approach for ^212^Pb-based therapy was introduced by Li *et al.* in 2021 88. They coupled the radionuclide lead-212 to a carrier vehicle consisting of calcium carbonate (CaCO_3_) microparticles (MP) for the treatment of disseminated disease in bodily cavities such as the peritoneal cavity.

##### Preclinical research

The stability of [^212^Pb]Pb-CaCO_3_-MP was confirmed after i.p. injection in tumor-free mice as limited redistribution to the kidneys, spleen, and liver was noted. Treatment of immunocompromised ES-2 ovarian cancer-bearing mice with [^212^Pb]Pb-CaCO_3_-MP using i.p. administration resulted in a significant survival benefit, which appeared to be dependent on amount of injected activity. However, in this study i.p. injection of unbound [^212^Pb]Pb-Cl_2_ also produced some therapeutic effect 88​.

Alternative carriers have been discussed in the review by Scaffidi-Muta and co-workers 13. In such cases, lead-212 is incorporated into large constructs such as colloids, fullerene nanoconstructs, liposomes, and other types of nanoparticles. As an example, fullerenes have been explored in the early 2000s 150,151. Liposomes have also been formulated 152,153 but were not translated into *in vivo* studies. In a previous literature review, we discussed the pitfalls for translation of alpha-radionuclide containing nanotechnology 154. The first major hurdle remains the lack of large-scale production methods that allow precise manufacturing at scale in compliance with GMP requirements. The second obstacle is that the size of these nanotechnologies must be strictly controlled to minimize splenic, hepatic and pulmonary accumulation, thereby reducing toxicity risks. Regulatory challenges of nanoparticles for medical application are a well-established hurdle and have been reviewed in detail 154. Finally, formulation in nanoparticles might change the toxicity profile of the radionuclides, and this also warrants more precise and cost-intensive investigations 154.

## Prospects and Future Directions for Lead-212 and Lead-203

Lead-212 has gained considerable interest in the evolving field of TAT. TAT is highly cytotoxic, delivering high LET over a short range in biological tissue, leading to double-stranded DNA breaks, complex chromosomal aberrations, and direct killing of cells by destruction of cellular components other than the DNA which also affects cell survival. These characteristics make TAT ideal for the treatment of minimal residual tumors or micro-metastatic disease. Moreover, unlike beta emitters, alpha emitters are less dependent on the oxidation state of malignant cells. Therefore, alpha emitters may offer a therapeutic alternative, specifically when beta-minus emitters become ineffective due to the development of radioresistance. Lead-212 is valuable for TAT because it functions as an *in vivo* alpha-particle generator, decaying into bismuth-212. During its decay both beta and alpha radiation is emitted, though the energy contribution of this beta particle is negligible compared to the alpha particle at the currently used clinically administered activities.

Key advantages of lead-212 for TAT include its relatively short half-life, which allows for maximum energy deposition in the cancer cells, while also reducing the burden of radioactive waste, yet still allowing for feasible production 3,12. Moreover, its diagnostic partner, lead-203, can be used for SPECT imaging, making it a versatile tool for theranostic applications. It is currently believed that lead-212 does have a realistic chance to be used on a large scale in nuclear medicine within the next decade 3,12.

### Production, availability and position among alpha emitters

Lead-212 is produced through various methods, all involving the separation of the radionuclide from thorium-228, radium-224, and various radionuclides throughout a complex decay chain. Advantages of lead-212 include logistics, due to the availability through either the form of ^212^Pb(HNO_3_)_2_ eluate (with a reasonable physical half-life of 10.6 hours) or in various generator systems based on a column separation or gaseous radon-220 emanation for the decentralized delivery of lead-212. More than 15 companies across Europe, North America, Asia, and Oceania are involved in the drive to promote lead-212 for preclinical validation and clinical applications 3,12.

The availability of the cyclotron produced lead-203 as a SPECT imaging agent allows for accurate dosimetry planning and can be an optimal theranostic partner for lead-212 during preclinical development and early clinical trials. Currently, the widespread clinical implementation of lead-203 as a theranostic derivative is not foreseen. This is due to the expensive infrastructure required for the cyclotron production of lead-203. Other routine theranostic partners could still include gallium-68 and fluorine-18 based tracers 3,4.

Several trials have validated the applicability of lead radioisotopes in a clinical setting. However, within TAT one radionuclide is unlikely to fit each clinical need. Lead-212 thereby complements other promising radionuclides such as actinium-225 and astatine-211 within the alpha emitter landscape. For example, the shorter half-life of lead-212 (10.6 h) compared to actinium-225 (~10 days) may be beneficial for reducing off-target toxicities, while actinium-225 leads to a higher absorbed dose (through four consecutive alpha-emissions) and hence higher damaging capacity per decay which may be beneficial in some indications. However, in both cases a crucial component is the retention of the alpha-emitting daughter radionuclides at the target site 155. In contrast to astatine-211, which is cyclotron-produced and requires centralized production, facing logistical challenges, lead-212 can be sourced from generator systems, offering a solution to availability in certain areas 53.

### Radiochemistry, chelation and dosimetry

A key factor in advancing lead-212-based therapies is the availability of robust chelators. Currently, TCMC is the best commercially available candidate and has demonstrated the highest *in vivo* stability for lead-212 chelation to date. However, many novel chelators are under active development. However, a disadvantage for lead-212, compared to therapies that are not based on *in vivo* generator radionuclides, is that the efficacy of the alpha emitter requires the radiopharmaceutical to remain stable upon beta decay. Otherwise, chemical instability may cause the release of the alpha emitting daughter, bismuth-212, which tends to accumulate in the kidneys 156. Furthermore, even though the effect of recoil is of lower magnitude with beta minus emissions compared to alpha emissions, it may still lead to the release of a percentage of bismuth-212 upon decay. Indeed, more optimal chelators need to be developed, or quick accumulation and strong trapping in the target is needed to allow the full deposition of the alpha emission in the tumor 3,30.

Diagnostic quantification of dosimetry with lead-203 will not consider the kidney exposure due to bismuth-212 that is released, and therefore, dosimetry with lead-212 remains necessary for accurate kidney dose estimation, as lead-203 studies do not account for bismuth-212 release. Since the stability of ^203^Pb- and ^212^Pb-radiopharmaceuticals can differ significantly, direct comparisons require careful evaluation 29,83,157.

### Vector optimization

It is essential to match the pharmaceutical half-life of lead-212 to the biological half-life of the vector to maximize the total amount of energy deposited in the tumor and minimize off-target irradiation upon systemic administration. Given the relative short half-life of lead-212, vectors with intermediate to fast pharmacokinetics are preferred, including antibody fragments, peptides or even small molecules. However, other vectors, such as full-length antibodies or inorganic microparticles, can also find their place in lead-212 based TAT through localized administration or pretargeting strategies, altering the biological distribution of the vector 3,53.

### Safety, toxicological and regulatory challenges

Despite the therapeutic promise, several safety and operational challenges remain. The high-energy gamma-ray emitted by thallium-208 during decay of lead-212 should also be considered to allow for radiation safety during radiopharmaceutical production, dispensing and administration 3,34. When working with lead-212 generators in the clinic, it is important that the consideration of radon-220 during generator use is investigated. In a recent study by Pretze and co-workers, the emanation system from CERN and the wet-chemical system from Perspective Therapeutics was evaluated with respect to radon-220 release. It was found that measurable radon-220 was only released during generator elution and not storage, and that the emanation-based generators are more prone to release than the wet-chemical system generators 46.

Moreover, the short half-life of lead-212 can cause high dose depositions *in vivo* at a relatively high dose rate, without requiring prolonged long retention (> 5 days). This can be advantageous when the radionuclide localizes within the tumor; however, it also poses a risk to healthy organs, such as the kidneys, where unintended accumulation may occur. Therefore, pharmacokinetics of each radiopharmaceutical construct need to be carefully optimized using accurate dose estimation tools and dosimetric models 53.

To ensure batch-to-batch consistency of each lead-212 or lead-203 radiopharmaceutical, regulations on Good Manufacturing Practice (GMP)-compliant production chains need to be implemented as well as reliable Quality Assurance protocols that relate to primary standards. Other logistical hurdles include a well-coordinated supply and distribution of the radiopharmaceutical, especially for lead-212, given its relatively short half-life 3. Examples of GMP-complaint production methods are available in literature 38,48.

### Radiobiological research

Research towards lead-212-based TAT has been explored using various targeting vectors as discussed in this review. This radiobiological research should take into account the pharmacological half-life of such vector molecules relating to the half-life of lead-212. However, altering the administration route of the TAT may overcome any discrepancies but should be extensively evaluated using proper biodistribution studies. Fractionated or repeated administration of lead-212 based therapy can also be explored. Even though the direct killing effect of alpha emitters may not be increased using repeated administrations, benefit may come from the increased chance of complete tumor eradication following repeated injections. Moreover, fractionating the therapy can be advantageous for reducing off target toxicity.

In addition, besides the direct cytotoxic effect of TRT, indirect effects should also be further explored using rationally designed preclinical studies. Such effects including the abscopal and bystander effects can only be explored in immunocompetent mice models, while current models used in the field are predominantly immunocompromised.

### Clinical trial design and patient selection

The design of clinical trials is vital for a successful translation of lead-212-based therapies. Multicenter, prospective, controlled studies with coordinated protocols are needed to generate high-quality, reproducible results that can lead to registration by EMA, FDA and other authorities and incorporation in clinical guidelines. Patient selection should be biomarker-driven, including theranostic imaging and all clinically relevant criteria, including prior treatments, should be considered.

### Next-generation strategies: pretargeting and combination therapies

Irradiation of healthy tissues and long systemic circulation related to the size of the vector molecule may be reduced through separation of the targeting and *in vivo* radiolabeling using a pretargeting approach. Herein, the non-bound vector is administered first, allowing tumor accumulation and clearance from the systemic circulation before administration of the radiopharmaceutical, which has a strong and rapid interaction with the target-bound vector molecule. Early-stage preclinical studies have shown promising results using this strategy for lead-212 based TAT 148,149.

Further, TAT may gain additional therapeutic efficacy when rationally combined with other treatment modalities. For example, TAT has been reported to stimulate the immune system through immunogenic cell death, triggering systemic responses that enhance therapeutic efficacy 10,158. This indicates potential synergy with immunotherapy such as immune checkpoint inhibitors 158. Moreover, the impact of TAT on DNA damage suggests potential benefit from combinations with other treatments influencing the DNA damage or repair such as PARP-inhibitors or chemotherapy. Early-stage research into these types of combinations is ongoing 158.

## Conclusions

Lead-212 and lead-203, represent promising radioisotopes for TAT and diagnostic imaging, respectively. Accumulating preclinical and clinical data support the therapeutic potential of lead-212 across multiple cancer types. Its theranostic counterpart, lead-203, plays a valuable role in the translational phase, enabling optimization of treatment schedules and dosimetry for lead-212-based therapies. Continued progress in radiochemistry, vector development, and patient stratification is expected to further expand the clinical impact of these isotopes. Lead-based radiopharmaceuticals are well positioned to play a central role in the future of precision oncology.

## Figures and Tables

**Figure 14:**
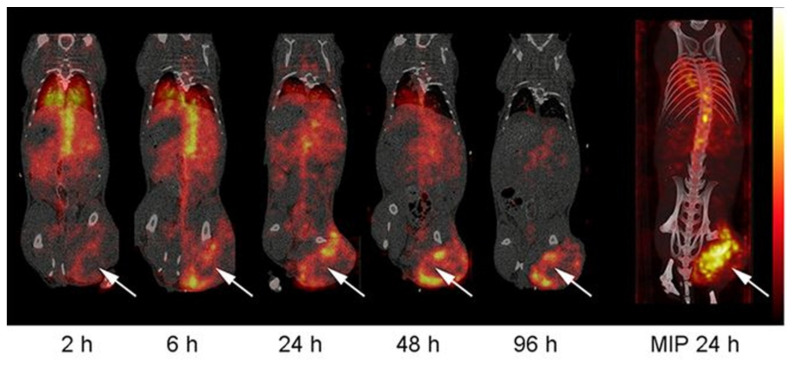
Serial SPECT/CT images of the biodistribution of [^203^Pb]Pb-TCMC-daratumumab targeting CD38 in RPMI8226 (multiple myeloma) bearing mice ​(Reprinted with permission, 140)​.

**Table 1:** A summary of the characteristics of reported lead-212 generators (grouped by parent and arranged chronologically):

Parent radionuclide	Matrix	Eluant	Manipulation of eluate	Yield	Ref
*Column generator systems based on thorium-228*					
Thorium-228 (37 MBq)	Na_2_TiO_3_ resin D-50 resin	Elute resin 1 with H_2_O, resin 2 with 2 M HCl	No manipulation is described	85% maximum yield	31
Thorium-228, loaded in 4 M HNO_3_	UTEVA resin SR resin prefilter resin	^212^Pb recovered from Sr resin with 10 mL 6 - 12 M HCl	No manipulation is described	>95%	32
Thorium-228, loaded in 40 mL of 1 M HNO_3_ stock solution	Pb resin column	1 mL of 1 M NH_4_OAc	No manipulation of the eluate is required	69.3 ± 4% yield	4
Thorium-228, loaded in 2 M HNO_3_ solution (22.9 MBq)	Pb resin Dowex 1 x 8 resin	Elute resin 1 with 2 M HCl and resin 2 with 0.01 M HCl	pH adjusted with NH_4_OAc	First elution 89% recovery; second elution 97% recovery	23
*Column generator systems based on radium-224*					
Radium-224, up to 925 MBq	AG-MP50 cationic exchange resin	2 M HI	The acid is neutralized with 1 M sodium acetate for further chemistry	90% yield	35
Radium-224	Cationic exchange resin Dowex-50X8-100 resin Dowex-1X-200 resin	3 mL of 2 M HCl	The eluate was left in a fume hood for radon-220 to dissipate and passed through an additional column to remove radium-224 breakthrough	Not provided	40
Radium-224, 0.15 MBq	Dowex 50W-x8 column	1 M HCl	No manipulation is described	Reported as quantitative	36
Radium-224		2 M HCl	Evaporation, then 3 digestions with 8 M HNO_3_, reconstitution with HNO_3_ 0.1 M and neutralization with 5 M NH_4_OAc	Not provided	14
Radium-224, up to 740 MBq loaded	AG-MP50 cationic exchange resin	4 mL of 2 M HCl	Evaporation of eluant, 3 repeat digestions with 8 M HNO_3_, extraction with 0.1 M HNO_3_, and neutralization with 5 M NH_4_OAc	>90%	28
Radium-224	AG-MP50 resin, Pb resin	Resin 1 eluted with 2 M HCl followed by resin 2 eluted with 5 M NH_4_OAc	No further manipulation is needed	86% yield from column 1 and 75.4% yield from column 2 (46.6 MBq/ml)	47
*Emanation generators based on capturing gaseous radon-220 [from either thorium-228 or radium-224*					
Thorium-228 (20-30 MBq)	Radon-220 gas collected in removable polyethylene bottle	Water (70% efficiency), 1 M NaCl (99% efficiency)	No manipulation is described	50 to 11% based on the age of the generator (tested to 1 year)	44
Thorium-228, up to 45 GBq	Radon-220 gas collected into a glass bubbler	HNO_3_ solution	No manipulation is described	70% yield (up to 25 GBq per day)	33
Thorium-228 (7.05 MBq)	^228^Th on Dowex ion exchange resin. Radon-220 gas is collected in a helix shaped vessel.	0.1 M HCl	No manipulation is described	40% collection efficiency from Dowex resin.	45
Radium-224 or thorium-228	Quartz wool containing parent, glass (Duran) flask to catch radon-220	Flask washed with 0.5 - 1 mL 0.1 M HCl	pH adjusted with NH_4_OAc or sodium acetate	Glass collector trapped 62 - 68% lead-212, eluted with >87% efficiency.	2
Radium-224	Duran-flask-method (CERN generator)	Flask rinsed 1-4x 1mL 0.1M HCl	Pb resin, eluted with NaAc/AcOH and purification after radiolabeling C18 column	Maximum recovery rate 31.6 ± 2.7%	46
Radium-224	VMT-α-GEN (PerspectiveTx) Non-FDA-approved	Elution with 4 mL 2 M HCl and 1 mL H_2_O	Pb resin, eluted with NaAc/AcOH and purification after radiolabeling on C18 column	Maximum recovery rate 76 ± 9%	48

**Table 2:** A summary of all clinical trials (active or completed) investigating lead isotope-based theranostics (clinicaltrial.gov - Sept 2025). Grouped by target and listed consecutively by trial start date.

Radioconjugate	Target	Patient population	Procedure	Source of Lead-212	Principle findings	Study identifier and dates
*Small molecules*						
[^212^Pb]Pb-NG001	PSMA	Patients with progressive, PSMA-expressing mCRPC (n=3)	Phase 0 and 1: Gamma camera imaging was performed at 1-3h and 16-24h p.i. of 9.4 MBq conjugate	Artbio	Feasible gamma counting and favorable dosimetry	**NCT05725070** [2023]
[^212^Pb]Pb-ADVC001	PSMA	Patients with PSMA-expressing mCRPC	Phase 1 and 2: Dose escalation and expansion study administering up to 200 MBq conjugate i.v. in 4-6 cycles, two/four weeks apart	Advancell - based on thorium-228	Not available	**NCT05720130** [2023-2029]
[^212^Pb]Pb-Pentixather	CXCR4	Patients with CXCR4-expressing atypical lung carcinoids	Early Phase 1: Two treatment cycles, 6 weeks apart in combination with [^203^Pb]Pb-Pentixather SPECT imaging pre and post-therapy (3 months)	University of Iowa - lead-212 source not disclosed	Not yet available	**NCT05557708** [2024-2028]
[^212^Pb]Pb-PSV359	FAP	Patients with FAP-expressing solid tumors	Phase 1 and 2: Dose escalation and expansion study with 92.5 - 370 MBq of iv administered radioconjugate	Perspective technology - based on radium-224	Not yet available	**NCT06710756** [2025-2032]
*Peptide-based vector*						
[^212^Pb]Pb-DOTAM- TATE	SSTR	Patients with SSTR-expressing NETs (n=20)	Phase 1: Dose escalation (1.13 - 2.50 MBq/kg/cycle) of i.v. administrated conjugate	Oranomed - based on radium-224	Well-tolerated	**NCT03466216** [2018-2022]
		Patients with SSTR- expressing NETs (n=66)	Phase 2: IV. injection of 2.5 MBq/kg/cycle conjugate for 4 cycles	Oranomed - based on radium-224	Preliminary results: ORR of 47.2%	**NCT05153772** [2021 - 2026]
[^203^Pb]Pb-VMT01	MC1R	Patients with MC1R-expressing metastatic melanoma	Phase 1: SPECT/CT images were obtained at 1h, 4h, and 24h p.i. of 555 - 925 MBq conjugate	Perspective technology - based on radium-224	Dosimetry successfully performed on SPECT/CT scans	**NCT04904120** [2021-2022]
[^212^Pb]Pb-DOTAM- GPR1	GRPR	Patients with recurrent or metastatic GRPR-expressing tumors	Phase 1: Dose escalation and expansion study with 3+3 design, dose increments of ±30%, four cycles eight weeks apart in MAD regimen	Oranomed - based on radium-224	Not yet available	**NCT05283330** [2022-2025]
[^203^Pb]Pb-VMT-ɑ-NET	SSTR	Patients with SSTR-expressing NETs	Early Phase 1: SPECT/CT images were obtained at 1h, 4h, 24h, and 48h p.i. of 185 MBq conjugate	University of Iowa - lead-212 source not disclosed	Dosimetry performed on SPECT/CT scans enabled dose selection for cohort 1 of NCT0614836	**NCT05111509** [2022-2025]
[^212^Pb]Pb-VMT-ɑ-NET	SSTR	Patients with SSTR-expressing NETs	Early Phase 1: Dose escalation study with up to two i.v. injections of conjugate at least eight weeks apart, co-administered with reno-protective amino acids	University of Iowa - lead-212 source not disclosed	Not yet available	**NCT06148636** [2023-2027]
		Patients with SSTR-expressing NETs	Phase 1 and 2: Dose escalation with four treatment cycles, every eight weeks including reno-protective amino acids	Perspective technology - based on radium-224	Not yet available	**NCT05636618** [2023-2029]
		Patients with SSTR-expressing NETs	Phase 1: Dose escalation, 4 treatment cycles every eight weeks, dosimetry - 3 year follow-up	National Cancer Institute - lead-212 source not disclosed	Not yet available	**NCT06479811** [2025-2029]
		SSTR-expressing NETs, pheochromocytomas, or paragangliomas	Phase 1 and 2: Dose escalation, 4 treatment cycles every eight weeks in a re-treatment setting (previous PRRT)	National Cancer Institute - lead-212 source not disclosed	Not yet available	**NCT06427798** [2025-2039]
[^212^Pb]Pb-VMT01	MC1R	Patients with MC1R-expressing metastatic melanoma	Phase 1 and 2: i.v. injection of 111-555 MBq conjugate in monotherapy or in combination with nivolumab	Perspective technology - based on radium-224	Not yet available	**NCT05655312** [2024-2029]
*Antibody-based vector*						
[^212^Pb]Pb-TCMC- trastuzumab	HER2	Patients with HER-2 expressing peritoneal metastases (n=18)	Phase 1: IV. injection of 4 mg/kg trastuzumab followed by i.p. injection of 7.4-27.4 MBq/m^2^ conjugate	Oranomed - based on radium-224	Acceptable pharmacokinetics, minimal distribution outside the peritoneal cavity, and limited toxicity	**NCT01384253** [2011 - 2016]

GRPR: gastrin-releasing peptide receptor, HER2: human epidermal growth factor receptor 2, MAD: multiple ascending dose, mCRPC: metastatic castrate resistant prostate cancer, MC1R: melanocortin 1 receptor, PRRT: peptide receptor targeting radioligand therapy. PSMA: prostate-specific membrane antigen, SSTR: somatostatin receptor, i.v.: intravenous, i.p.: intraperitoneal, p.i.: post-injection, ORR: objective response rate, N/A: not applicable

## Abbreviations

BB2: Bombesin receptor subtype 2
CAFs: Cancer-associated fibroblast
CERN: Conseil européen pour la Recherche nucléaire
CXCR4: C-X-C motif chemokine receptor 4
DNA: Deoxyribonucleic acid
DOTA: 1,4,7,10-tetraazacyclododecane-1,4,7,10-tetraacetic acid
DOTAM: 1,4,7,10-tetraazacyclododecane-1,4,7,10-tetraacetamide
EDTA: Ethylenediaminetetraacetic acid
EGFR: Epidermal growth factor receptor
FAP: Fibroblast activating protein
FDA: United States Food and Drug Administration
GPRP: Gastrin-releasing peptide receptor
HCl: Hydrochloric acid
HDEHP-teflon: Di-(2-ethylhexyl) phosphoric acid
HER2/1: Human epidermal growth factor receptor 2/1
HNO_3_: Nitric acid
i.p.: Intraperitoneal
keV: Kiloelectronvolt
LET: Linear energy transfer
MC1R: Melanocortin-1 receptor
mCRPC: Metastatic castrate-resistant prostate cancer
MeV: Megaelectronvolt
MP: Microparticles
NaI: Sodium iodide
NET: Neuroendocrine tumors
PSC: Lead-specific chelator
PET: Positron emission tomography
PSMA: Prostate-specific membrane antigen
PTK-7: Protein tyrosine kinase 7
RNT: Radionuclide therapy
SPECT: Single-photon emission computed tomography
SSTR: Somatostatin receptors
TAT: Targeted alpha therapy
TCMC: 1,4,7,10-tetraazacyclododecane-1,4,7,10-tetraacetamide
TRT: Targeted radionuclide therapy
UTEVA: Uranium and TEtraValents Actinide Resin
VCAM: Vascular cell adhesion molecule
